# Isolation and Characterization of the Polyvalent Enterobacteria-Infecting Phage Cit2 with Potential for Biocontrol Applications

**DOI:** 10.1021/acsomega.5c10441

**Published:** 2026-02-25

**Authors:** Paloma Cavalcante Cunha, Isabella Ribeiro Rodrigues, Ana Julia Dill Rosseto, Jéssica Duarte da Silva, Marcella Silva Vieira, Roberto Sousa Dias, Cynthia Canêdo da Silva, Sérgio Oliveira de Paula

**Affiliations:** † Department of Microbiology, 28120 Federal University of Viçosa, Avenida Peter Henry Rolfs, s/n, Viçosa 36570-900, Minas Gerais, Brazil; ‡ Department of General Biology, Federal University of Viçosa, Avenida Peter Henry Rolfs, s/n, Viçosa 36570-900, Minas Gerais, Brazil

## Abstract

*Salmonella enterica* is a major foodborne pathogen globally, often associated with poultry and fresh produce. The rising prevalence of multidrug-resistant (MDR) strains and the limited efficacy of conventional decontamination methods highlight the need for alternative, targeted strategies. Bacteriophages (phages) have emerged as promising biocontrol agents due to their specificity, safety, and potential for direct application in food systems. In this study, we isolated and characterized the polyvalent lytic phage *Citrobacter* phage vB_CfrD-Cit2 (Cit2) and evaluated its effectiveness against *S. enterica* in two food matrices: chicken meat and lettuce. Cit2 belongs to the *Tequintavirus* genus and showed lytic activity against *Citrobacter freundii*, *Shigella flexneri*, and different *S. enterica* serovars. Genomic analysis confirmed the absence of virulence, antibiotic resistance, or lysogeny-related genes, supporting its classification as genetically safe. The phage remained stable across a broad range of pH values and temperatures. In biocontrol assays, Cit2 significantly reduced *S. enterica* serovar Enteritidis counts on lettuce at room temperature by 1.83, 1.84, and 1.55 log_10_ CFU/mL after 1, 2, and 24 h, respectively. In refrigerated chicken meat (4 °C), reductions of 0.79, 0.84, 0.76, and 0.69 log_10_ CFU/mL were observed at 1, 6, 24, and 48 h post-treatment, respectively. Phylogenetic and protein identity analyses suggest that Cit2 likely targets the outer membrane receptor FhuA, similarly to phage T5. Future studies should aim to optimize multiplicity of infection (MOI) and cocktail formulations to improve host range and efficacy, as well as assess phage stability across different food matrices. Taken together, these findings support Cit2 as a promising candidate for the development of phage-based interventions to enhance food safety.

## Graphical abstract

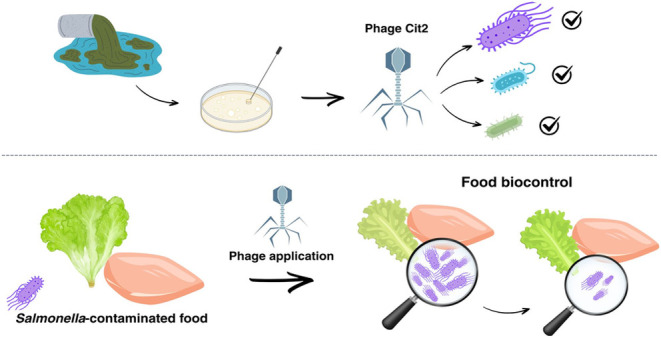

## 1 Introduction

Nontyphoidal *Salmonella* (NTS) is one of the leading causes of foodborne illnesses worldwide, responsible for millions of cases of gastrointestinal infection annually and a substantial burden of hospitalizations and deaths in vulnerable populations.^1^ In Brazil and elsewhere, *Salmonella* remains one of the most frequently reported agents in foodborne outbreaks, with several serovars—notably *S.* Enteritidis and *S.* Typhimurium—repeatedly implicated in human disease.^2,3^ These bacteria colonize a wide range of animal reservoirs and food matrices (including poultry, eggs, pork and fresh produce), and can persist at multiple points along the food production chain (from farm to fork), making control efforts particularly challenging.^4–7^ The emergence and dissemination of multidrug-resistant (MDR) *Salmonella* strains—driven by the widespread use of antibiotics in human and veterinary medicine—have further complicated treatment and containment, underscoring the urgent need for improved strategies to reduce contamination and human exposure.^1,8–10^ Contamination can occur at multiple stages throughout the food production chain, including animal farming, slaughter, processing, transportation, retail, and even during food handling. Poultry meat—particularly chicken—is one of the most common sources of *Salmonella* contamination.^7,11^ To mitigate its spread, several preventive and control strategies are employed, including good agricultural and manufacturing practices, microbiological monitoring, cleaning and disinfection protocols, and hazard analysis and critical control point (HACCP) systems.^12,13^ In Brazil, microbiological criteria for food safety are established by the Brazilian Health Regulatory Agency (ANVISA) through Normative Instruction No. 161/2022, which mandates the absence of *Salmonella* in all food products.^14,15^ Similar regulations exist in other countries, such as the United States and the European Union.^16,17^ Despite these standardized sanitation efforts, *Salmonella* remains difficult to eliminate and persist in poultry production systems.^7^

Current food decontamination strategies rely on thermal treatments, chemical sanitizers, and nonthermal physical technologies. Thermal processes such as pasteurization and sterilization are effective but can negatively affect sensory and nutritional quality, which limits their applicability for fresh or minimally processed foods.^18^ Chemical sanitizers—especially chlorine-based compounds—remain widely used, yet their antimicrobial activity is reduced in the presence of organic matter, they may generate potentially harmful disinfection byproducts, and repeated exposure can contribute to microbial tolerance within biofilms.^19–21^ Nonthermal technologies, including high-pressure processing (HPP), ultraviolet (UV) light, pulsed electric fields, ozone, and cold plasma, preserve “fresh-like” characteristics but show variable efficacy depending on the food matrix, limited penetration, or require high operational and equipment costs.^18,22–24^ Together, these limitations reveal important gaps in current food safety systems, particularly for controlling surface-associated or biofilm-protected *Salmonella* in poultry and fresh produce.^25,26^

Bacteriophages complement conventional decontamination approaches because they are highly specific to bacterial targets and can be applied directly to contaminated surfaces or food matrices with minimal impact on organoleptic properties.^27^ In the food industry, phage applications have traditionally focused on two major strategies: oral administration to reduce *Salmonella* colonization in the gastrointestinal tract of poultry and surface disinfection of food products and processing equipment during postharvest stages.^28,29^ These interventions help prevent cross-contamination and significantly reduce pathogen loads in poultry products.^30^ Phages offer several advantages over chemical sanitizers and other antimicrobials, including high specificity, self-amplification at the site of infection, and compatibility with direct use in complex food matrices.^31–33^ Importantly, phage treatment does not alter the visual, textural, or sensory quality of foods, making them suitable not only for pathogen reduction but also for biopreservation strategies aimed at extending shelf life.^34–36^ In addition, a broad host range makes phages particularly appealing for industrial use, as polyvalent phages can expand the spectrum of bacterial targets in phage formulations and allow propagation in nonpathogenic strains, thereby minimizing the risk of cross-contamination with the target pathogen during production.^33,37^

Numerous studies have demonstrated the efficacy of phage-based interventions in reducing *Salmonella* contamination across a variety of food products. Commercial formulations such as SalmoFresh and Salmonelex have proven effective in meats, vegetables, and eggs, highlighting their potential as practical tools in food safety management.^31,38^ Beyond these practical advantages, phages are especially effective against biofilm-associated or surface-persistent bacteria that frequently survive conventional sanitizers.^21^ They also integrate well into multihurdle strategies, such as sequential application with washing steps, HPP, or mild organic acids, providing enhanced reductions in pathogen levels.^27,39^ Limitations associated with phage use—including potential narrow host range, emergence of phage-resistant bacterial variants, environmental stability, and regulatory constraints—are being addressed through strategies such as phage cocktails, immobilization and encapsulation methods, and careful genomic screening to exclude temperate or virulence-associated genes^27–29,40,41^

Taken together, these characteristics position phage biocontrol not as a replacement but as a targeted, residue-free, and industrially adaptable complementary technology that fills critical gaps left by existing decontamination methods, thereby underscoring the relevance and novelty of developing and characterizing new polyvalent phages, such as the *Citrobacter* phage vB_CfrD-Cit2 (Cit2) investigated in this study. Building on this need, we characterized Cit2 and demonstrated that it is a robust and genomically safe lytic phage active against *Salmonella enterica*, evaluating its host range, genomic features, physicochemical stability, and biocontrol performance in relevant food matrices.

## 2 Materials and Methods

### 2.1 Bacterial Strains and Growth Conditions

*Citrobacter freundii* ATCC 8090 was used as the isolation host for *Citrobacter* phage vB_CfrD-Cit2, hereafter referred to as Cit2. All bacterial strains used in this study are listed in Table 1 and were employed to investigate the host range of the phage. *Escherichia coli* strains were kindly provided by Dr. Maria Aparecida Moreira, Laboratório de Doenças Bacterianas of the Universidade Federal de Viçosa (UFV), Viçosa, Brazil. *Serratia marcescens* was kindly provided by Dr. Maria Cristina Vanetti, Laboratório de Microbiologia Industrial, UFV. Some of the *S. enterica* subs. *enterica* strains were isolated from mesenteric lymph nodes of pigs and kindly provided by Dr. Ricardo Yamatogi, Departamento de Medicina Veterinária, UFV, while others were isolated from poultry farming and kindly provided by a partner company.

**Table 1:** Host Range of Phage Cit2^a^,^b^

strain	source	spot test	average title (PFU/mL)	EOP
*C. freundii* ATCC 8090	TC	+	5.9 × 10^8^	host
*Shigella flexneri* ATCC 12022	TC	+	1 × 10^9^	1.7
*S.* Enteritidis ATCC 13076	TC	+	1.5 × 10^9^	2.5
*S.* Typhimurium	swine	+	3.0 × 10^8^	0.5
*S.* 1,4,[5],12:-:1,2	swine	+	1.6 × 10^6^	0.002
*S.* Panama	swine	+	2.8 × 10^8^	0.47
*S.* Infantis	swine	+	0	0
*S.* Derby	swine	+	0	0
*S.* Cerro	swine	–		
*S.* Heidelberg 63623	poultry (BC)	+	0	0
*S.* Heidelberg 65499	poultry (SH)	+	0	0
*S.* Minnesota 64303	poultry (BC)	–		
*S.* Minnesota 65374	poultry (BB)	–		
*S.* Mbandaka 64166	poultry (FF)	–		
*S.* Mbandaka 64188	poultry (FF)	–		
*E. coli* ATCC 29214	TC	+	0	0
*E. coli* K12	TC	+	0	0
*E. coli* 304	HH	+	0	0
*E. coli* 30	BM	+	0	0
*E. coli* 4	BM	–		
*E. coli* 20	BM	–		
*E. coli* CDC0111ab	TC	–		
*E. cloacae* ATCC 13047	TC	–		
*S. marcescens* MIND01	environmental	–		

a Bacterial strains were tested by spot assay for susceptibility to infection. Efficiency of plating (EOP) was determined only for strains that produced visible growth inhibition in spot tests. EOP values were categorized as follows: high (≥0.5), moderate (0.2 to <0.5), low (0.001 to <0.2), and no plaque formation (0). “+” indicates visible growth inhibition in spot assay; “–” indicates no visible alterations.

b TC = type collection; poultry: BC = broiler chicken, BB = broiler breeder, FM = feed mill, SH = slaughterhouse; BM = bovine mastitis; HH = human hospital.

The strains were cultivated in Luria–Bertani (LB) broth (10 g/L of NaCl_2_, 10 g/L of peptone, and 5 g/L of yeast extract) at a temperature of 37 °C. Xylose Lysine Deoxycholate (XLD) agar (Kasvi, Curitiba, Paraná, Brazil) was used to identify and differentiate *Salmonella* in food tests. The optical density (OD) of the cultures was measured on a spectrophotometer (Shanghai Spectrum SP-1105, Shanghai, China) at a wavelength of 600 nm (OD_600_) to infer the phase of bacterial growth.

### 2.2 Phage Isolation and Propagation

Phage Cit2 was isolated from urban sewage samples collected in Viçosa (Minas Gerais, Brazil). For the isolation, the Twest and Kropinski^42^ enrichment protocol was carried out. Briefly, the samples were centrifuged at 10,000*g* and 4 °C for 10 min, and the supernatant was filtered through a 0.22 μm PES membrane (Millipore, Billerica, MA). Then, 5 mL of sterile double-strength LB broth plus 2 mM CaCl_2_ (0.22 g/L) was inoculated with 0.1 mL of a *C. freundii* culture (Table 1) in logarithmic growth phase (OD_600_ ∼ 0.5) and mixed with 5 mL of the filtered sewage sample. The mixture was incubated at 37 °C and 100 rpm for approximately 24 h. After that, the mixture was centrifuged at 10,000*g* and 4 °C for 10 min and the supernatant (lysate) was filtered (0.22 μm membrane). The lysate was then subjected to the double-layer agar (DLA) technique^42,43^ and incubated overnight.

Among the resulting lysis plates, one plaque was selected, excised, and subjected to a double-layer agar method for further propagation. This procedure—plaque picking followed by replating—was repeated at least three times to ensure the isolation of a single, clonal phage population. The purified phage was then propagated in LB broth supplemented with 2 mM CaCl_2_, following the protocol described by Sambrook and Russell,^44^ titrated as previously reported,^43^ and stored at 4 °C. For experiments involving *S.* Enteritidis, the phage was also propagated using this bacterial host.

### 2.3 Host Range

The ability of Cit2 to infect different bacterial strains was initially assessed by spotting 10 μL of the phage suspension onto the surface of double-layer agar plates previously inoculated with the test bacteria (Table 1). Plates were incubated overnight at 37 °C. Bacterial strains that exhibited clear zones or lysis plaques at the spot site were considered susceptible to the phage and were selected for subsequent efficiency of plating (EOP) assays.

To determine the host range and more accurately evaluate productive infection, the EOP was calculated as described by Khan Mirzaei and Nilsson.^45^ Briefly, phage lysates were serially diluted and plated on susceptible strains using the DLA technique. Plates were incubated overnight at 37 °C, and plaque-forming units per milliliter (PFU/mL) were determined the following day. The EOP was calculated by dividing the average PFU/mL obtained on the test strain by the average PFU/mL obtained on the original host strain. EOP was classified as high (≥0.5), moderate (0.2–0.49), low (0.001–0.199), or inefficient (≤ 0.001), following Khan Mirzaei and Nilsson.^45^

### 2.4 Morphology Analysis

Phage morphology was analyzed by transmission electron microscopy (TEM). The phage suspension was first concentrated and purified by ultracentrifugation over a 20% sucrose cushion at 15,000 rpm for 7 h at 4 °C. After centrifugation, the supernatant was discarded, and the pellet was resuspended in 100 μL of ultrapure water. A 10 μL aliquot of this suspension was applied to the Formvar-coated grids. After 5 min, excess liquid was removed with filter paper, and the sample was negatively stained with 2% (w/v) uranyl acetate for 15 s. Grids were left in a desiccator for 24 h before imaging with a Zeiss EM 109 transmission electron microscope (Zeiss, Oberkochen, Germany) at the Center for Microscopy and Microanalysis (UFV).

Capsid and tail dimensions were measured using three independent micrographs of distinct phage particles, and average values were calculated using ImageJ version 1.54g software (National Institutes of Health, Bethesda, MD).

### 2.5 One-Step Growth Curves

One-step growth curves were performed to determine the burst size and latent period of phage Cit2 in *C. freundii* ATCC 8090 and *S.* Enteritidis ATCC 13076, following the protocol described by Niu et al.^46^ Briefly, 10 mL of host bacterial culture at approximately 10^8^ CFU/mL was infected with phage suspension at a multiplicity of infection (MOI) of 0.0001. The mixture was incubated at 37 °C for 10 min to allow phage adsorption and then centrifuged at 10,000*g* for 10 min to remove unadsorbed phages. The pellet containing infected cells was resuspended in 10 mL of LB broth, and a 100 μL aliquot was immediately collected to determine the initial phage titer (time zero).

The culture was incubated at 37 °C with shaking (100 rpm) for a total of 90 min. Samples of 100 μL were collected every 5 min for the first 40 min, and then every 10 min thereafter, and titrated using the DLA method. The latent period was defined as the time between the adsorption and the onset of the first burst. The burst size was calculated as the ratio between the final number of phage particles and the initial count.

### 2.6 Multiplicity of Infection Influence

To evaluate the influence of multiplicity of infection (MOI) on host bacterial growth, optical-density-based growth curves were performed using 96-well polystyrene microplates. Briefly, phage Cit2 was diluted in SM buffer (5.8 g/L NaCl, 2.0 g/L MgSO_4_·7H_2_O, 50 mL of 1 M Tris-HCl, 5 mL of 2% gelatin; pH 7.5), and 20 μL of the phage suspension was added to 180 μL of bacterial culture in early exponential phase (OD_600_ = 0.1, approximately 1.5 ×  10^7^ CFU/mL) in LB medium, to achieve final MOIs of 0.1, 1, and 10. Control wells received 20 μL of SM buffer instead of phage. Microplates were incubated at 37 °C in a Multiskan GO spectrophotometer (Thermo Scientific), and bacterial growth (OD_600_) was recorded every 15 min over a 21 h period.

### 2.7 Thermal and pH Stability

Stability assays were conducted to assess the potential applications of phage Cit2 under different food processing and storage scenarios. The phage was evaluated for pH and thermal stability following the protocol described by Sváb et al.,^47^ with minor modifications. In all cases, the final phage concentration was adjusted to 1 × 10^7^ PFU/mL. Immediately after treatment, phage preparations were diluted in SM buffer, plated using the DLA method, and titrated. All assays were performed in triplicate.

For pH stability testing, 10 μL of phage suspension was added to 990 μL of LB broth adjusted to various pH values (ranging from pH 2 to pH 12) and incubated at 25 °C for 2 h. LB broth (pH 7) served as the control. For thermal stability, phage suspensions in LB broth (pH 7.0) were incubated for 48 h at −20 °C, for 2 h at 25, 40, 50, 60, and 70 °C, and for 5 min at 80 and 90 °C. A nonheated aliquot kept at 4 °C was used as the control.

### 2.8 *In Vitro* Phage Challenge at Low Temperature

A bacterial culture of *S.* Enteritidis at OD_600_ = 0.4 (approximately 5 × 10^8^ CFU/mL) was diluted in LB broth to a final concentration of 1 × 10^5^ CFU/mL. Subsequently, 100 μL of the Cit2 phage lysate was added to 9.9 mL of this bacterial suspension to achieve multiplicities of infection (MOIs) of 100 and 1000. Control tubes received 100 μL of SM buffer instead of phage. All tubes were incubated at 4 °C, and culture samples were collected at 0, 1, 3, 5, 24, and 48 h postinoculation. At each time point, aliquots were serially diluted in phosphate-buffered saline (PBS; 137 mM NaCl, 2.7 mM KCl, 10 mM Na_2_HPO_4_, 1.8 mM KH_2_PO_4_; pH 7.4), plated on LB agar using the spread plate method, and incubated overnight at 37 °C. Bacterial counts (CFU/mL) were determined the following day.

### 2.9 Biocontrol of *S.* Enteritidis in Food

#### 2.9.1 Biocontrol in Fresh Lettuce

This assay was based on the methodology described by Spricigo et al.,^48^ with modifications. *S.* Enteritidis was grown in LB broth until mid log phase (OD_600_ = 0.4, approximately 5 × 10^8^ CFU/mL). The culture was centrifuged at 9000 rpm for 15 min, the supernatant was discarded, and the bacterial pellet was resuspended in 10 mL of 0.9% NaCl sterile solution. The suspension was then diluted to a final concentration of 1 × 10^5^ CFU/mL. Phage Cit2 was diluted in 0.9% NaCl to a final titer of 1 × 10^8^ PFU/mL. Fresh lettuce was washed under running tap water and surface-disinfected by immersion in a sodium hypochlorite solution (200 ppm) for 15 min. The leaves were then rinsed with sterile distilled water to remove the residual chlorine.

To contaminate the surface with *S.* Enteritidis, the lettuce leaves were immersed in the bacterial suspension prepared before for 5 min at room temperature, then transferred to sterile Petri dishes and allowed to dry under aseptic conditions for approximately 15 min. The dried leaves were cut into smaller sections, and 5 g was weighed into each Petri dish to serve as an individual replicate. Samples were then immersed in 10 mL of the phage solution and incubated for 10 min. Control samples were treated similarly but received phage-free 0.9% NaCl instead. A negative control group, consisting of lettuce leaves that were not inoculated with either bacteria or phage, was included to confirm the effectiveness of the hypochlorite disinfection procedure.

After treatment, the leaves were transferred to new sterile Petri dishes and left to dry for an additional 15 min. Once dried, the samples were stored at room temperature (∼25 °C) in sterile tubes. Samples were collected at 0, 1, 2, and 24 h post-treatment. To recover bacteria, 2 mL of 0.9% NaCl was added to each sample and gently agitated for 5 min to dislodge bacterial cells. The resulting suspension was plated on XLD agar using the spread plate method and incubated at 37 °C overnight. The number of viable *Salmonella* cells (CFU/mL) was determined the following day.

#### 2.9.2 Biocontrol in Chicken Meat

This assay was adapted from the methods described by Sukumaran et al.^38^ and Pelyuntha and Vongkamjan,^49^ with modifications. The preparation of *S.* Enteritidis inoculum and phage Cit2 solution was performed as described in the previous section (Biocontrol in fresh lettuce).

Chicken breast fillets were purchased from a local supermarket and aseptically cut into ∼2 × 2 cm^2^ cubes. The meat pieces were then immersed in a 50 ppm chlorine solution, prepared with autoclaved distilled water, for 5 min to reduce background microorganisms. To remove residual chlorine, each meat piece was sequentially transferred through three separate beakers containing sterile distilled water, remaining immersed for 5 min in each. Excess water was removed, and the meat pieces were air-dried in the biological safety cabinet for 15 min at room temperature.

Each side of the disinfected meat cube received 50 μL of the bacterial suspension (100 μL total per piece) and was left undisturbed for 5 min to allow bacterial attachment. Excess liquid was removed, and the contaminated pieces were transferred to new Petri dishes and left to dry for an additional 15 min. Then, 50 μL of the phage suspension was surface-applied to each side (100 μL total per piece). Control groups received the same volume of sterile saline instead of phage solution. After 10 min of phage exposure, each meat piece was placed into an individual sterile tube and stored at 4 °C.

Samples were collected at 0, 1, 6, 24, 48, and 72 h post-treatment. To recover bacteria, 2 mL of PBS was added to each tube, and samples were agitated for 5 min to release attached bacterial cells. The resulting liquid was serially diluted and plated on XLD agar using the spread plate method. Plates were incubated at 37 °C for 24 h, and typical black-centered *Salmonella* colonies were enumerated. A negative control group, consisting of meat pieces not inoculated with either bacteria or phage, was included to confirm the absence of *Salmonella* after disinfection with the 50 ppm chlorine solution.

### 2.10 Genomic Characterization and Phylogenomic Analysis

#### 2.10.1 Phage DNA Isolation and Sequencing

Phage genomic DNA was extracted using the PCI/SDS protocol available at PhagesDB (PCI/SDS DNA Extraction 2.2013, http://phagesdb.org). Briefly, 1 mL of high-titer phage lysate (≥10^9^ PFU/mL) was treated with MgCl_2_ (1 M, 12.5 μL), DNase I (2000 U/mL, 0.4 μL), and RNase A (100 mg/mL, 1 μL), followed by incubation at room temperature for 30 min. Subsequently, EDTA (0.5 M, 40 μL), Proteinase K (10 mg/mL, 5 μL), and SDS (10%, 50 μL) were added, and the sample was incubated at 55 °C for 1 h. DNA was then purified through two rounds of extraction with an equal volume of phenol/chloroform/isoamyl alcohol (25:24:1), followed by gentle mixing and centrifugation at 12,000*g* for 5 min. The upper aqueous phase was recovered and precipitated with 95% ethanol (1 mL) and sodium acetate (3 M, 50 μL), and incubated on ice for 5 min. After centrifugation at 12,000*g* for 10 min and a wash with 70% ethanol, the DNA pellet was air-dried and resuspended in nuclease-free water or TE buffer. DNA quality was assessed by spectrophotometry and agarose gel electrophoresis.

Sequencing was performed on the Illumina NovaSeq 6000 platform by Novogene Bioinformatics Technology Co., Ltd. (Davis, CA). Raw read quality was assessed with FastQC (version 0.11.9; https://github.com/s-andrews/FastQC). Adapter sequences were removed using TrimGalore (version 0.6.7; default settings), and further trimming was conducted with Trimmomatic (version 0.36) using the parameters HEADCROP:10, CROP:140, SLIDINGWINDOW:4:20, and MINLEN:130. High-quality reads were assembled *de novo* using SPAdes (version 3.15.4), with all odd K-mer sizes between 21 and 99. Assembly quality was assessed using Assembly-Stats (version 1.0.1), and contigs shorter than 1000 bp were excluded. Candidate phage contigs were identified based on size and coverage, and confirmed by BLASTn alignment against the NCBI nucleotide database.

#### 2.10.2 Genome Annotation, Analysis, and Taxonomic Assessment

Prediction and initial annotation of open reading frames (ORFs) were performed using the RAST server (https://rast.nmpdr.org/rast.cgi) in May 2021. Each predicted ORF was subsequently manually curated using the BLASTp algorithm at NCBI (https://blast.ncbi.nlm.nih.gov/BlastAlign.cgi) and the InterProScan web service (https://www.ebi.ac.uk/interpro/) to generate the final consensus annotation table and to search for possible protein domains. Additional *in silico* analyses were performed to assess genomic features of interest. The presence of putative tRNA genes was evaluated using the tRNAscan-SE tool (Galaxy Version 0.4) via the Phage Galaxy server (https://phage.usegalaxy.eu/). Screening for antimicrobial resistance genes and virulence factors was carried out using ResFinder v4.7.2 ^50–52^ and VirulenceFinder v2.0.5,^53,54^ both hosted at the CGE web platform (https://cge.food.dtu.dk/). The visual genomic map of Cit2 was generated using the Proksee platform (https://proksee.ca/) and integrated into the annotation process to support the manual curation of predicted ORFs and structural feature identification.

To identify the closest relatives and determine the taxonomic placement of phage Cit2, its complete genome sequence was compared with the NCBI nucleotide database using the BLASTn tool. A randomized subset of RefSeq genomes from the *Markadamsvirinae* subfamily (taxonomy ID 2732013) was retrieved from the NCBI Virus database (https://www.ncbi.nlm.nih.gov/labs/virus/vssi/#/). The genome of Cit2 was then added to this data set and analyzed through the ViPTree server (https://www.genome.jp/viptree/) to construct a proteomic-based phylogenetic tree and infer evolutionary relationships. Intergenomic similarity was assessed using the VIRIDIC web tool (http://rhea.icbm.uni-oldenburg.de/viridic/), applying ICTV thresholds of 95 and 70% to delineate species and genus boundaries, respectively.

The complete genome of Cit2 was deposited in the NCBI GenBank in October 2022 and is available under accession number OP745948.1.

#### 2.10.3 Phylogenetic Analysis of Receptor-Binding Protein (RBP) and Llp Sequences

The amino acid sequence of the receptor-binding protein (RBP) and the superinfection exclusion protein (Llp) of phage Cit2, along with RBPs and Llps from *Markadamsvirinae* phages with known bacterial receptors, was aligned using MAFFT v7 at MPI Bioinformatics Toolkit (https://toolkit.tuebingen.mpg.de/tools/mafft) with the L-INS-i strategy. The phylogenetic tree was reconstructed using the Maximum Likelihood method implemented in MEGA 12. The LG model with γ-distributed rates among sites (LG + G) was applied, and gaps were treated with the “Use All Sites” option. Branch support was assessed by standard bootstrap analysis with 1000 replicates, and bootstrap values greater than 70% were considered indicative of a strong phylogenetic support. Trees were analyzed to assess the clustering of Cit2 relative to phages with experimentally characterized receptors (FhuA, BtuB, or FepA), enabling the inference of the most probable receptor used by Cit2 based on its phylogenetic proximity to functionally annotated phages.

### 2.11 Statistical Analysis

All experiments were performed in triplicate. Statistical analyses were conducted using GraphPad Prism software, version 8.3.0 (538). Data normality was assessed using the Shapiro–Wilk test prior to statistical analysis, and appropriate tests were selected based on the results. For growth curve comparisons, multiple unpaired *t* tests (one per time point) were performed, with statistical significance determined using the Holm–Sidák method and an α level of 0.05. Each time point was analyzed independently without assuming a consistent standard deviation (SD). For the stability assays, low-temperature infectivity tests, and food biocontrol experiments, unpaired test mixtures were used to compare the means between control and Cit2-treated groups for each condition.

## 3 Results

### 3.1 Phage Host Range and Morphology

The phage used in this study was isolated from *C. freundii* ATCC 8090 and named *Citrobacter* phage vB_CfrD-Cit2 (Cit2). The phage was able to reach high titers, exceeding 10^9^ PFU/mL, within a few hours (∼7 h) when propagated in this host, producing small (∼1 mm), translucent plaques on LB agar. The host range of Cit2 is listed in Table 1. Efficiency of plating (EOP) assays were performed only for strains that yielded positive results, i.e., growth inhibition (+) in spot tests. The results showed that Cit2 was able to infect representatives of three genera within the *Enterobacteriaceae* family. In addition to its original isolation host (*C. freundii*), Cit2 successfully infected different serovars of *S. enterica* (*S.* Enteritidis, *S.* Typhimurium, *S.* 1,4,[5],12:-:1,2, and *S.* Panama), as well as a strain of *S. flexneri* (ATCC 12022). Highly productive infections (EOP ≥ 0.5) were observed in *S.* Enteritidis, *S.* Typhimurium, and *S. flexneri*. In contrast, infection in *S.* Panama was classified as moderately productive (EOP 0.2 to <0.5), and infection in *S.* 1,4,[5],12:-:1,2 as low productivity (EOP 0.001 to <0.2).

Although Cit2 caused visible growth inhibition in some *E. coli* and *Salmonella* strains in the spot test, no lysis plaques were detected by the double-layer agar assay (DLA), suggesting that the observed effects may be attributed to mechanisms other than productive infection, such as lysis from without. Future assays quantifying the phage titer (PFU/mL) in these cultures should be performed to determine whether phage replication is occurring.

Microscopy analysis revealed that phage Cit2 has an isometric head measuring 55 nm in diameter and a long, thin, flexible tail approximately 200 nm in length (Figure 1A). One-step growth curves were performed using the original isolation host (*C. freundii*) and the host with the highest efficiency of plating (*S.* Enteritidis). The results showed that both the latency period and burst size of Cit2 varied between the two hosts: the latency period was 30 min in *C. freundii* (Figure 1B) and 25 min in *S.* Enteritidis (Figure 1C), while the burst size was 434 PFU/cell in *C. freundii* and 100 PFU/cell in *S.* Enteritidis.

**Figure 1:**
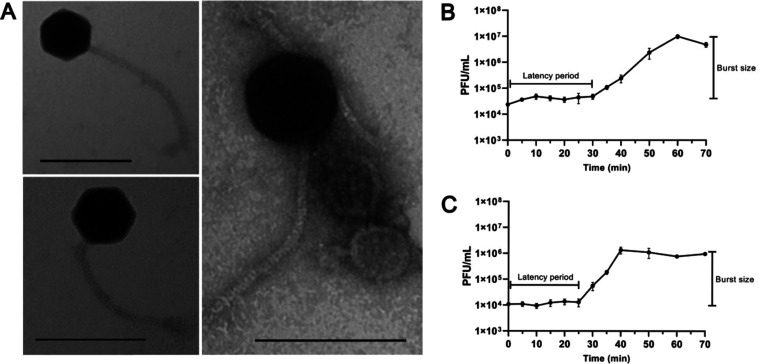
Transmission electron microscopy (TEM) image of phage Cit2, with the scale bar representing 100 μm (A). One-step growth curves of the phage in its original isolation host *C. freundii* (B) and in the host exhibiting the highest efficiency of plating (EOP), *S.* Enteritidis (C).

### 3.2 Genome Analysis and Taxonomic Assessment

#### 3.2.1 Genome Analysis

The Cit2 phage has a double-stranded DNA genome of 111,610 bp with a G+C content of 39.1%. A total of 146 putative ORFs were identified, of which 58 were predicted to encode functional proteins and 88 were annotated as hypothetical proteins of unknown function. Additionally, 21 tRNA-encoding genes were found, with one remaining unclassified (Table 2). Although the genome topology (linear or circular) was not experimentally determined, the genome map was represented as circular (Figure 2) for visualization purposes. Functional ORFs were grouped into seven categories based on their predicted roles: DNA, RNA, and nucleotide metabolism; auxiliary metabolic genes and host takeover; head and packaging; connector; tail; lysis; and other functions. The genomic map also depicts tRNA genes, GC content, and GC skew profiles.

**Figure 2:**
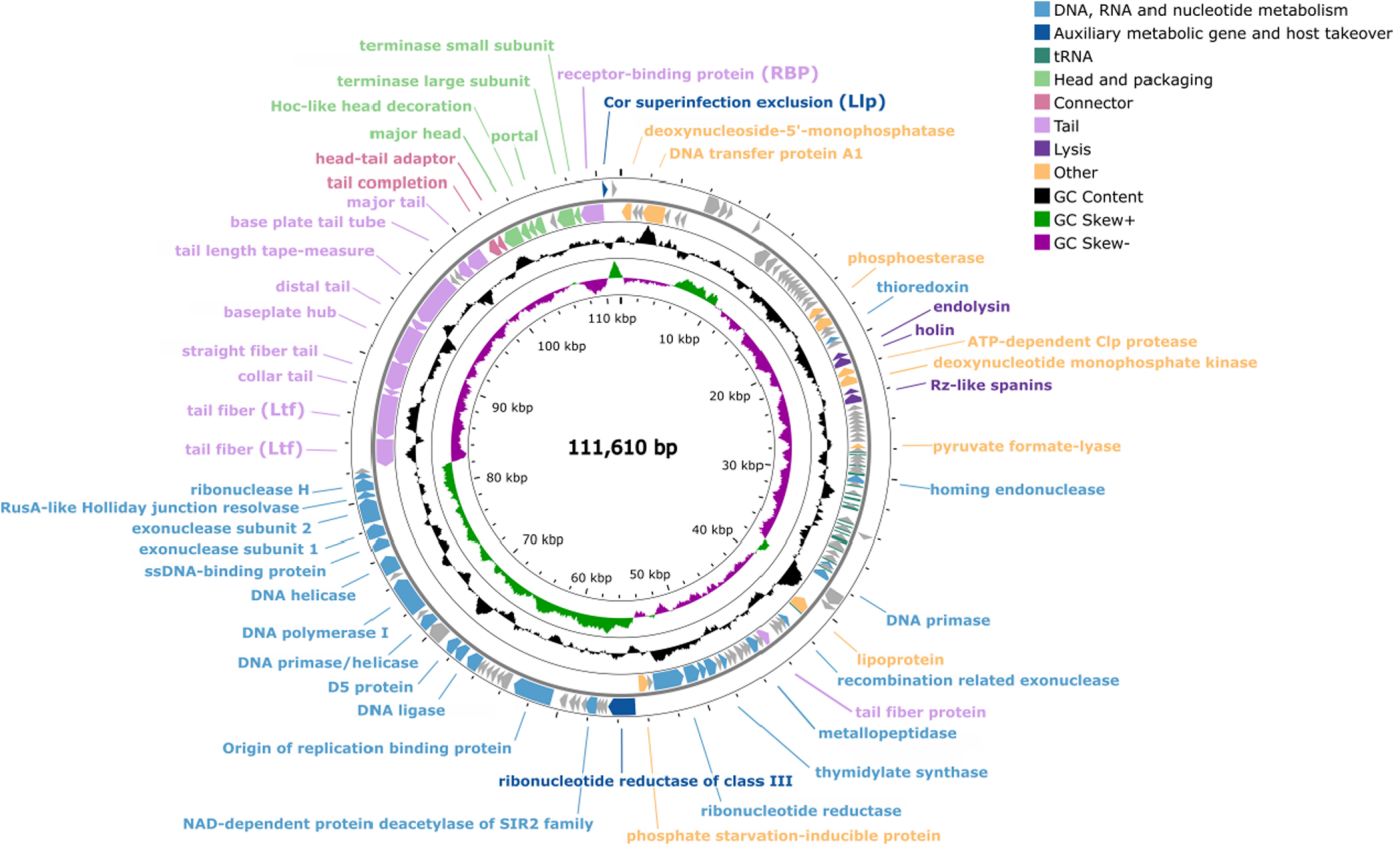
Circular genome map of phage Cit2 generated using Proksee, based on functional annotation performed with RAST. Each gene is color-coded according to its predicted functional module. Genes encoding hypothetical proteins with unknown function are shown in gray.

**Table 2:** Transfer RNA (tRNA) Genes Predicted in the Genome of Phage Cit2 Using tRNAscan-SE^a^

tRNA	anti-codon	begin	end	cove score
Undet		41738	41667	21.25
Arg	TCT	41164	41093	45.87
Ser	GCT	37453	37368	60.60
Met	CAT	37358	37284	40.78
Leu	TAA	36774	36701	60.37
Tyr	GTA	35760	35683	46.56
Glu	TTC	35130	35059	55.65
Cys	GCA	34531	34459	46.27
Asn	GTT	34303	34224	56.07
Lys	CTT	33663	33591	70.94
Pro	TGG	32597	32523	60.52
Met	CAT	32513	32439	36.75
Lys	TTT	32246	32171	62.14
Ala	TGC	31882	31811	56.52
Leu	TAG	31802	31729	50.60
Ser	TGA	31516	31430	22.77
Gln	CTG	30002	29930	61.83
Gln	TTG	29920	29846	62.85
Thr	TGT	29325	29242	65.44
Ile	GAT	28488	28415	68.17
Met	CAT	28315	28243	51.74

a The table lists the corresponding amino acid, anti-codon, genomic position, and prediction score.

Among the structural genes, proteins involved in host recognition and adsorption—key determinants of host range—were identified. A receptor-binding protein (RBP), commonly responsible for binding to specific bacterial receptors,^55^ was predicted in ORF 144. ORF 145 encodes an *Llp*-type receptor-blocking protein, which has been associated with superinfection exclusion mechanisms in T5-like phages.^56,57^ Two L-shaped tail fiber proteins (Ltf), known to participate in initial adhesion and potential host range expansion,^58,59^ were annotated in ORFs 124 and 125. No genes related to antibiotic resistance, virulence factors, or lysogeny were detected. A complete list of all predicted ORFs, including their annotations and genomic positions, can be accessed in the GenBank entry for Cit2 (accession number: OP745948.1).

#### 3.2.2 Taxonomic Assessment and Phylogenetics

The BLAST search revealed that the genome of phage Cit2 shares high sequence identity with *Salmonella* phage oldekolle (query cover 90%; identity 96.86%; accession number NC_048865.1) and *Salmonella* phage S131 (query cover 92%; identity 96.72%; accession number NC_048009.1), both members of the genus *Tequintavirus* (order *Caudovirales*, family *Demerecviridae*, subfamily *Markadamsvirinae*). In the proteomic tree based on genome-wide sequence similarities among members of the *Markadamsvirinae* subfamily, phage Cit2 clustered with other phages of the *Tequintavirus* genus (Figure 3), revealing close similarity relationships with members of this group. Consistent with the BLAST and proteomic tree analyses, VIRIDIC analysis (Figure 4) also showed high similarity with *Tequintavirus* phages, with the highest similarity scores obtained for phage NBSal003 (RefSeq accession: NC_048856.1, similarity score: 89.6), *Salmonella* phage S131 (RefSeq accession: NC_048009, similarity score: 89.4), and *Salmonella* phage oldekolle (RefSeq accession: NC_048865.1, similarity score: 88.0). Thus, our phage can be considered a representative of a novel species within the genus *Tequintavirus*.

**Figure 3:**
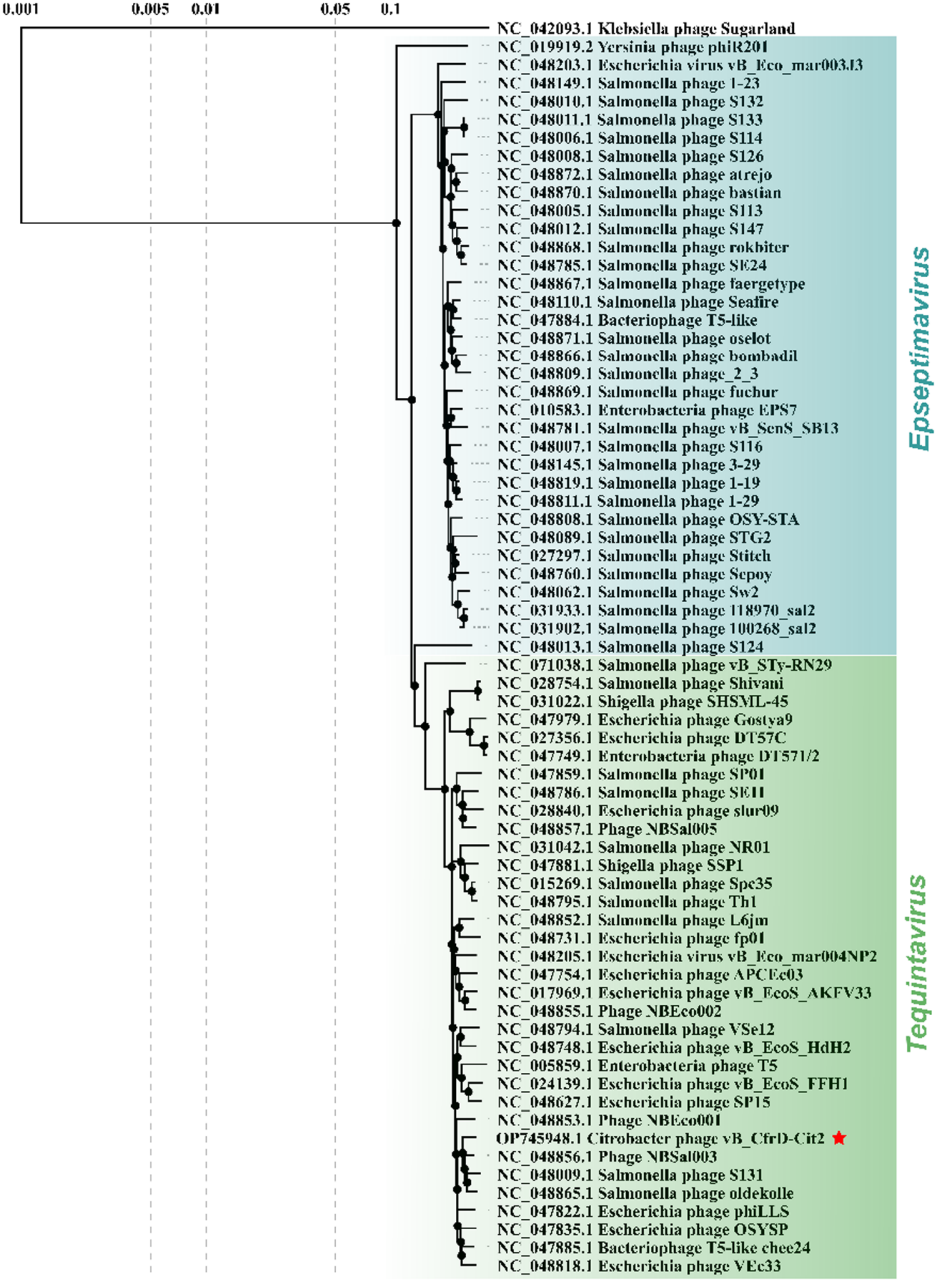
Genome-wide proteomic tree generated using VipTree for phage Cit2 (indicated by a red star) and reference genomes (RefSeq) of *Markadamsvirinae* subfamily members. The tree was constructed based on genome-wide amino acid sequence similarities calculated with tBLASTx, using the ViPTree server’s default settings.

**Figure 4:**
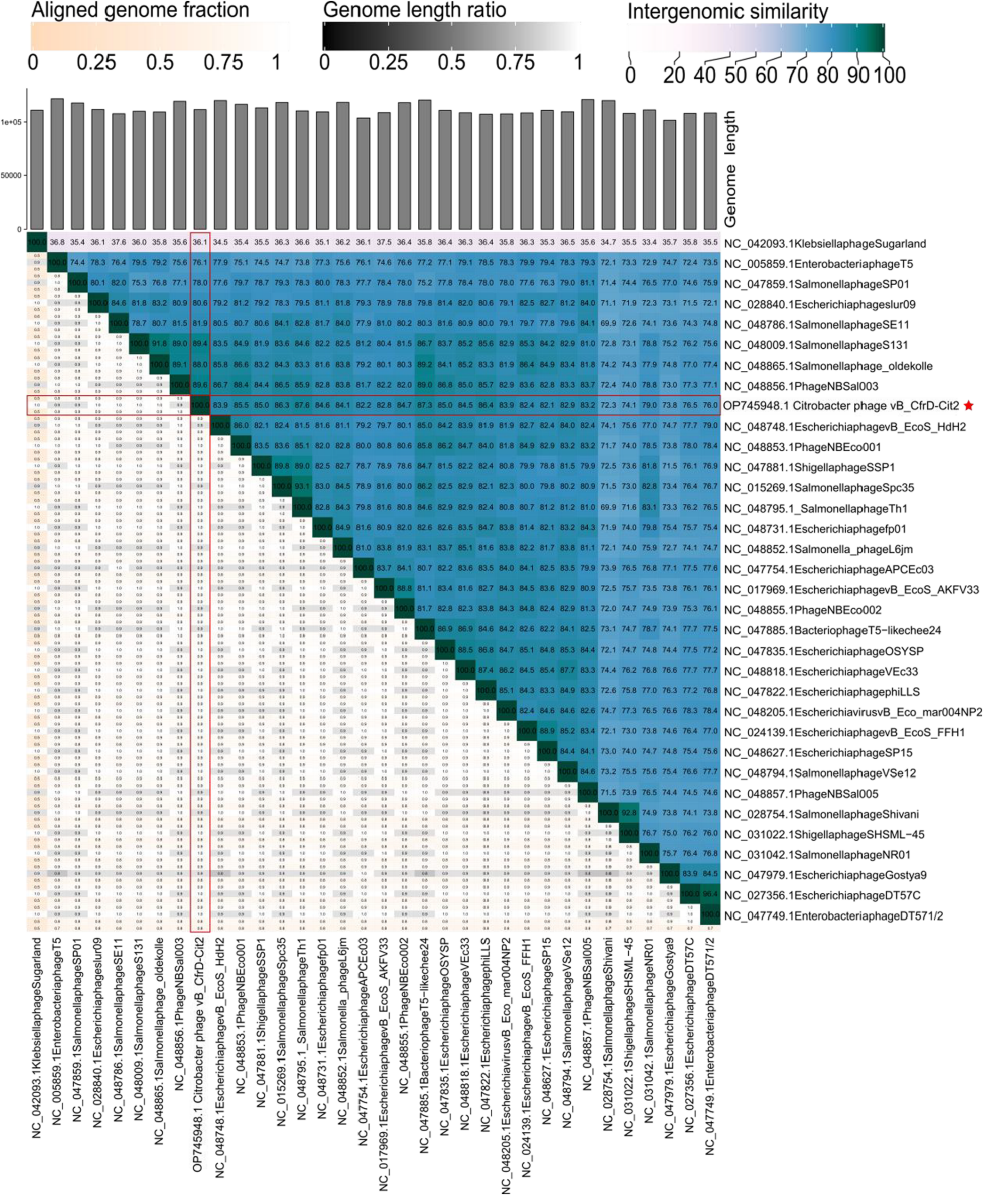
Intergenomic similarity matrix generated using VIRIDIC for phage Cit2 (indicated by a red star) and reference genomes (RefSeq) of *Markadamsvirinae* subfamily members. Pairwise nucleotide identity was calculated using BLASTn, and results are presented as a heatmap where darker shades indicate higher similarity.

The Clinker analysis of the complete genome (Figure 5) of Cit2, alongside phages from the *Markadamsvirinae* subfamily with known receptor usage, was performed to examine genome organization and globally aligned gene clusters. The phages included in the analysis were *Enterobacteria* phage *T5* (Accession number: NC_005859), *Salmonella* phage *Spc35* (Accession number: NC_015269), and *Salmonella* phage S124 (Accession number: NC_048013).

**Figure 5:**
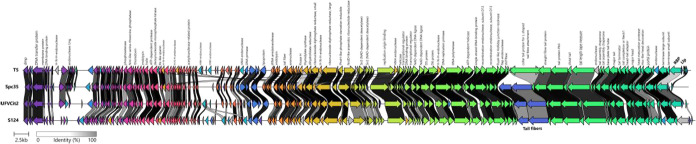
Genomic synteny analysis of phage Cit2 and reference genomes from the *Markadamsvirinae* subfamily with known receptor usage, generated using Clinker. Genes are automatically color-coded according to similarity groups. Gray shading between arrows indicates regions of amino acid similarity (threshold ≥30%). GenBank accession numbers: *Enterobacteria* phage T5 – NC_005859, *Salmonella* phage Spc35 – NC_015269, and *Salmonella* phage S124 – NC_048013.

Synteny analysis demonstrated a strong conservation of genes associated with essential biological functions, including DNA replication, virion assembly, and host lysis processes. Notably, almost all predicted structural proteins were highly homologous across the compared genomes with the exception of host recognition elements such as the receptor-binding protein (RBP) and the L-shaped tail fibers, which exhibited greater divergence. In contrast, regions enriched with hypothetical proteins and proteins of unknown function displayed considerable variability. These findings indicate that while core functional modules remain conserved among Tequintavirus genomes, variable regions likely represent accessory components contributing to the genomic plasticity observed among closely related *Tequintavirus* isolates.^60,61^

#### 3.2.3 Receptor Inference from RBP and Llp Clustering

To investigate the probable receptor recognized by Cit2, we first analyzed the amino acid identity between its receptor-binding protein (RBP) and those of *Markadamsvirinae* phages with known receptor usage. The RBPs of T5-like phages are typically located at the distal end of a straight tail fiber and are responsible for binding to specific outer membrane receptors. Depending on the phage, these receptors can include ferrichrome (FhuA), vitamin B12 (BtuB), or ferric enterobactin (FepA) transporters.^62–64^ In addition to receptor recognition during initial adsorption, some T5-like phages encode a small lipoprotein (Llp) expressed after genome injection, which binds to the receptor to prevent superinfection and protect progeny phages from being inactivated by infected cells.^56,57^

The RBP of Cit2 exhibited 96.9% percent identity with the RBP of phage T5, which recognizes FhuA as the bacterial receptor, but only 30.8% identity with the RBP of phage S131, which has also been proposed to target the same receptor.^59^ With BtuB-binding phages S126,^59^ Spc35,^65^ Bf23 ^61,62^ e DT57C,^66^ the RBP of Cit2 showed 67.0, 71.8, 66.7, and 73.2% identity, respectively. In contrast, the percent identity with the RBPs of phages H8 and S124, which recognize FepA,^59,63^ was only 27.7%.

To further explore these relationships, phylogenetic trees were constructed based on the amino acid sequences of the RBP and the superinfection exclusion protein Llp. In the RBP-based phylogeny (Figure 6A), phages grouped largely according to their experimentally confirmed receptor. BtuB-binding phages (S114, S133, S126, S116, Bf23, Spc35, and DT57C) formed a well-supported clade. Despite being a BtuB-binding phage, DT57C was positioned closer to the FhuA-binding group. Cit2 clustered strongly with phage T5 (bootstrap 99%), which uses FhuA as its receptor, supporting the hypothesis that Cit2 also recognizes FhuA. In contrast, phages H8 and S124 (FepA-binding) and phage S131 (previously proposed to use FhuA) formed separate clades, with S124 and H8 grouping together (bootstrap 100%), and S131 remaining isolated.

**Figure 6:**
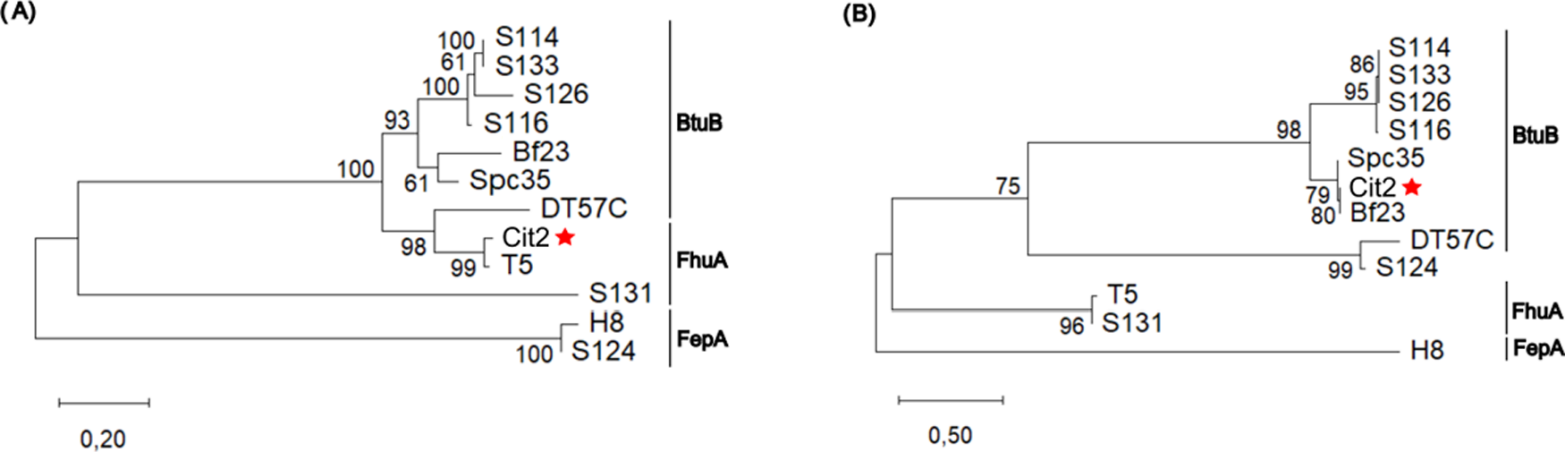
Maximum likelihood phylogenetic tree based on (A) receptor-binding protein (RBP) and (B) superinfection exclusion protein (Llp) sequences of Cit2 (indicated by a red star) and reference *Markadamsvirinae* phages with experimentally characterized or presumed host receptors. The tree was constructed using MEGA 12 with 1000 bootstrap replicates. Bootstrap values ≥50% are shown. The scale bar represents amino acid substitutions per site. The corresponding GenBank accession numbers were as follows: S114 (NC_048006), S133 (NC_048011), S126 (NC_048008), S116 (NC_048007), Bf23 (OR083247), Spc35 (NC_015269), DT57C (NC_027356), T5 (NC_005859), S131 (NC_048009), H8 (NC_042307), and S124 (NC_048013).

The phylogenetic tree based on Llp sequences (Figure 6B) revealed a partially different topology. In this tree, Cit2 grouped with the BtuB-binding phages Spc35 and Bf23 (bootstrap values of 79 and 80%, respectively), separating it from the T5/S131 clade (bootstrap 96%), with which it had clustered in the RBP tree. The BtuB-binding phages S114, S133, S126, and S116 formed a well-supported clade (bootstrap values ≥95%), while DT57C and S124 grouped in a closely related branch (bootstrap 99%). Notably, phages T5 and S131, both associated with FhuA recognition, formed a distinct clade (bootstrap 96%), whereas phage H8, which uses FepA as its receptor, was positioned as a distant outgroup.

Together, these results reinforce the inference that Cit2 utilizes FhuA as its primary receptor based on both its close phylogenetic relationship and high sequence identity to T5. The divergence observed in the Llp phylogeny, however, suggests possible divergence in Llp evolution among Markadamsvirinae members and may reflect adaptation to specific receptor-blocking mechanisms across different hosts.

### 3.3 Multiplicity of Infection (MOI) Influence

This and the subsequent assays were conducted by using *S.* Enteritidis as a target host to explore the biocontrol potential of phage Cit2 against this pathogen. The growth curves of *S.* Enteritidis in the presence of phage Cit2 demonstrated the influence of different multiplicities of infection (MOIs) on the bacterial proliferation (Figure 7). A statistically significant reduction in bacterial growth (*P* < 0.05) was observed in all phage-challenged groups compared to the phage-free control after 21 h, regardless of the MOI, confirming the lytic activity of Cit2 throughout the 21 h assay period.

**Figure 7:**
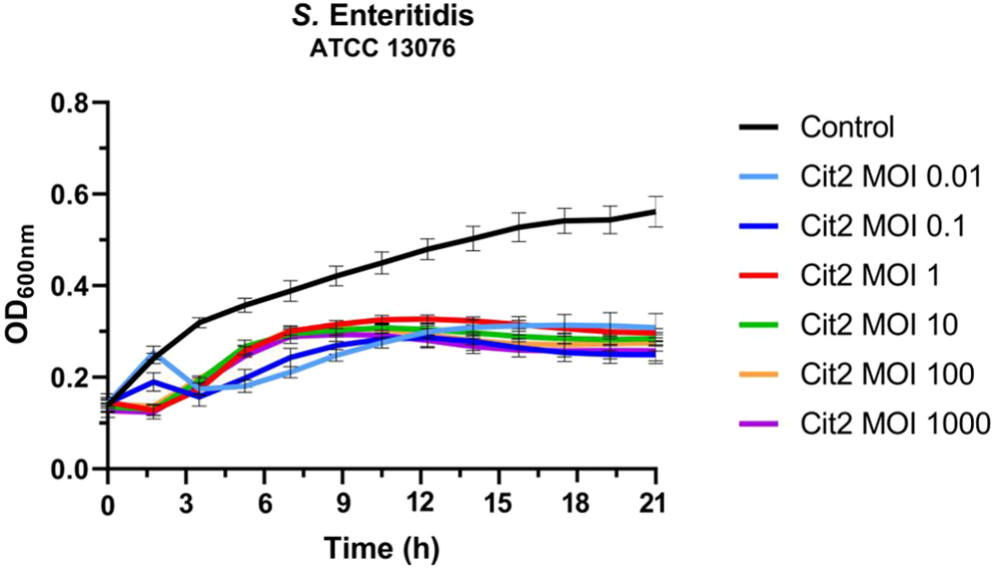
Bacterial growth curves of *S.* Enteritidis over 21 h in the presence of phage Cit2 at different multiplicities of infection (MOIs), ranging from 0.01 to 1000. Optical density (OD_600_) measurements were taken at regular intervals (15 min) to assess the impact of phage infection on bacterial growth dynamics. The bars on the different points of the curve line indicate the standard deviations of the mean.

At low MOIs (0.01 and 0.1), initial bacterial growth was detected during the first 1.5 h of incubation, followed by a sharp decline in optical density (OD). This was succeeded by a partial regrowth phase beginning after approximately 3 h. The growth curves stabilized around 10 h (MOI 0.1) and 13 h (MOI 0.01), maintaining constant OD values until the end of the experiment. At intermediate to high MOIs (1–1000), bacterial growth was initially suppressed, with no increase in OD (*P* > 0.05) observed during the early incubation period. However, exponential growth resumed around 7 h, after which OD values plateaued and remained relatively stable until the experiment concluded. This pattern suggests that phage Cit2 effectively restricted bacterial proliferation following an initial adaptation or escape phase.

Despite the early kinetic differences observed between MOIs, statistical analysis revealed no significant difference (*P* > 0.05) in final bacterial densities among the phage-treated groups after 21 h. These findings underscore the complex dynamics of phage-host interactions, in which early suppression is more pronounced at higher MOIs, while long-term outcomes may be modulated by factors such as bacterial regrowth, resistance development, or variability in phage replication efficiency.

### 3.4 Thermal and pH Stability

Aiming at future applications of phage Cit2 for the control of *S. enterica*, stability assays were performed using *S.* Enteritidis as the host bacterium. The tests revealed that the phage was highly stable at pH values ranging from 4 to 12 (Figure 8A) and at temperatures up to 60 °C (Figure 8B). However, a reduction of 3.38 log_10_ PFU/mL was observed at pH 3, and reductions of 0.24, 0.3, 4.46, and 5.90 log_10_ PFU/mL were recorded at −20, 60, 70, and 80 °C, respectively. Complete phage inactivation occurred at pH 2 and at 90 °C.

**Figure 8:**
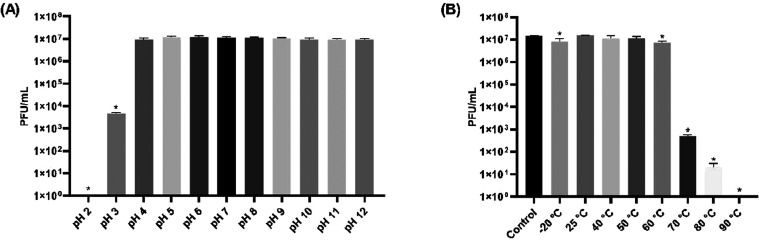
Stability of phage Cit2 under different environmental conditions. (A) pH stability assay conducted at room temperature for 1 h across a pH range of 2–12; pH 7 served as the control. (B) Thermal stability assay performed at various temperatures (from 4 to 90 °C) for 1 h; 4 °C was used as the control condition. Phage titers are expressed as log_10_ PFU/mL. Data represent mean values from independent experiments, with error bars indicating standard deviation. Statistically significant differences (*P* < 0.05) are indicated by asterisks (*).

### 3.5 *In Vitro* Phage Challenge at Low Temperature

This assay was designed to assess whether the phage Cit2 is capable of lysing *S.* Enteritidis at low temperatures, where bacterial replication—and consequently phage propagation—is strongly limited. To favor the possibility of “lysis from without,” in which bacterial cell lysis is induced externally without requiring phage replication inside the host,^67^ high MOIs (100 and 1000) were used. This strategy increases the chance of multiple phages adsorbing to a single bacterium, promoting direct lysis even in nonpermissive conditions for phage replication. Evaluating the phage activity at 4 °C is particularly relevant for its potential application as an antibacterial or biocidal agent in refrigerated food products.

Significant bacterial reductions (*P* < 0.05) were observed for both MOIs at all evaluated time points. For MOI 100 (Figure 9A), the reductions relative to the control were 1.13, 1.05, 1.23, 1.16, and 1.07 log_10_ CFU/mL at 1, 3, 5, 24, and 48 h, respectively. For MOI 1000 (Figure 9B), more pronounced reductions were observed: 2.35, 2.24, 2.25, 2.24, and 2.75 log_10_ CFU/mL at the same time points. These results indicate that Cit2 can effectively reduce *Salmonella* viability under refrigeration conditions, supporting its potential use in food safety applications.

**Figure 9:**
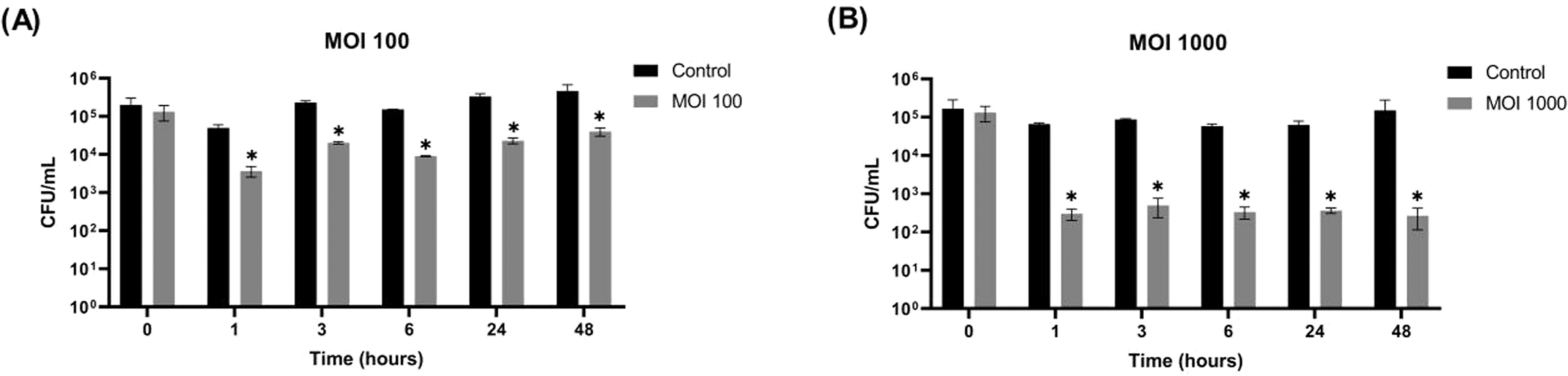
*In vitro* lytic activity of phage Cit2 at low temperature over a 48 h period. The assay was performed at multiplicities of infection (MOIs) of 100 (A) and 1000 (B), and bacterial growth was monitored to assess phage-mediated inhibition under refrigeration conditions.

### 3.6 Biocontrol of *S.* Enteritidis in Food

Treatment of lettuce leaves was performed at room temperature (∼25 °C), which reflects typical conditions used for the storage and transport of fresh produce. The 10 min phage immersion step was designed to simulate the common household practice of vegetable sanitization. A reduction of 1.83 log_10_ CFU/mL was observed after 1 h of phage treatment, followed by 1.84 log_10_ at 2 h, and 1.55 log_10_ at 24 h, compared to the untreated control (Figure 10A). These results demonstrate that phage Cit2 was able to significantly reduce *S.* Enteritidis loads on lettuce surfaces over time, even as the bacterial counts increased substantially in the control group.

**Figure 10:**
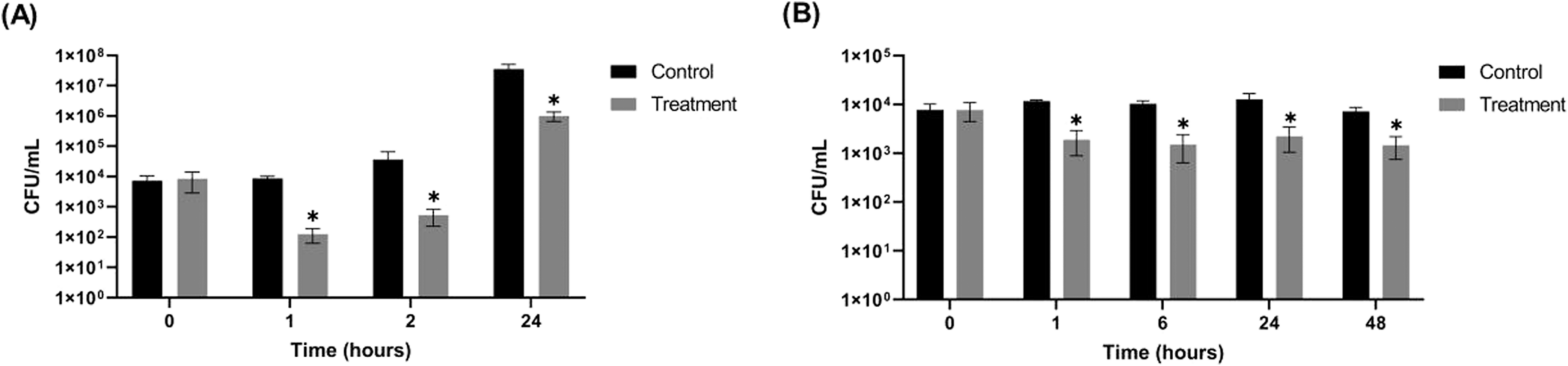
Biocontrol of *S.* Enteritidis using phage Cit2 at a multiplicity of infection (MOI) of 1000. (A) Reduction of bacterial counts on lettuce at room temperature. (B) Reduction of bacterial counts on chicken meat at 4 °C. Statistically significant differences (*P* < 0.05) are indicated by asterisks (*).

Chicken meat samples were treated with phage Cit2 and subsequently stored at 4 °C for 72 h to simulate postprocessing refrigeration conditions commonly used in the poultry industry. Although phage application is still primarily experimental, this approach was designed to mimic the potential integration of phage treatment into existing chilling and storage steps in poultry processing workflows. Significant reductions in *S.* Enteritidis counts were observed in the treated group compared to the untreated control (Figure 10B). Reductions of 0.79, 0.84, 0.76, and 0.69 log_10_ CFU/mL were observed at 1, 6, 24, and 48 h post-treatment, respectively. These results demonstrate that phage Cit2 was effective in maintaining a lower bacterial load during refrigerated storage, with a sustained reduction over the 48 h period.

Negative control samples (meat pieces not inoculated with *Salmonella* or phage) confirmed the absence of *Salmonella* in the product following immersion in the 50 ppm hypochlorite solution. However, some yellow and translucent colonies were observed on XLD plates, indicating the presence of background microorganisms and suggesting that the disinfection procedure was not fully effective against other bacteria. In *Salmonella*-inoculated samples, these nonblack colonies were present but remained scarce up to 48 h, allowing reliable enumeration of black *Salmonella* colonies. However, by 72 h, the overgrowth of nontarget bacteria rendered accurate quantification of *Salmonella* unfeasible.

## 4 Discussion

The increasing prevalence of multidrug-resistant (MDR) *S. enterica* serovars in food production chains has prompted the search for sustainable and effective alternatives to traditional antimicrobial agents. In this context, phages have emerged as promising biocontrol tools due to their specificity, self-replicating nature, and ability to target bacteria even under adverse environmental conditions.^31,68–70^ In the present study, we characterized the polyvalent lytic phage Cit2 and evaluated its potential application in controlling *S. enterica* in food matrices. Our results collectively highlight its effectiveness, genomic safety, host range breadth, and environmental resilience—key attributes for phage-based biocontrol strategies.

The phage Cit2 was isolated from sewage using *C. freundii* ATCC 8090 as the original host and demonstrated the ability to infect a broad spectrum of Enterobacteriaceae, including key pathogenic *S. enterica* serovars (Enteritidis and Typhimurium), strains isolated from poultry and swine production, and *S. flexneri*. Although Cit2 did not infect the *E. coli* strains tested in this study, it is important to note that the number of *E. coli* strains evaluated was limited. Therefore, we cannot rule out the possibility that Cit2 is capable of infecting other *E. coli* strains. Future studies should expand the *E. coli* panel in host range analyses to better assess this potential. Notably, high efficiency of plating (EOP ≥ 0.5) was observed in *S.* Enteritidis and *S.* Typhimurium, two of the most prevalent serovars associated with human salmonellosis.^4,5^ The ability to infect *S. flexneri* further reinforces its polyvalence—a highly desirable trait for phages intended for use in complex microbial communities such as those found in food-processing environments.^29,38^ Additionally, the successful propagation in the nonpathogenic biosafety level 1 strain *C. freundii* further highlights the feasibility of large-scale and safe production of Cit2 for industrial applications, minimizing the likelihood of unintentional contamination of production facilities with the target pathogen.

Importantly, Cit2’s genome lacks identifiable genes related to lysogeny, antibiotic resistance, or virulence—essential criteria for phage safety in food applications.^31^ The presence of 21 tRNAs suggests that this phage is equipped to optimize translation of its own proteins during replication, a feature often associated with increased lytic efficiency in large-genome phages.^71^ Characterization of Cit2 by electron microscopy revealed morphological features typical of the *Tequintavirus* genus within the Demerecviridae family, characterized by an isometric capsid and a long, flexible tail. Consistent with T5virus properties, the Cit2 genome demonstrated high sequence identity with known *Tequintavirus* genomes, particularly *Salmonella* phages Oldekolle and S131, supporting its taxonomic classification and enabling insights into phage-host interactions and host range determinants. Despite the high conservation of genes involved in virion morphogenesis and lytic functions, synteny and phylogenetic analyses revealed substantial variability in accessory regions and host recognition modules such as receptor-binding proteins (RBPs) and L-shaped tail fibers (Ltf). This variability likely contributes to the diverse host range observed among related phages and highlights the evolutionary flexibility of this group in adapting to distinct enterobacterial receptors.^61,63,66^

Functional inference based on the RBP phylogeny suggests that Cit2 likely utilizes FhuA, a TonB-dependent outer membrane receptor, as its primary bacterial receptor. This is supported by high amino acid identity (96.9%) and close phylogenetic clustering with phage T5, a canonical FhuA-binding phage.^55,72^ FhuA is involved in both iron uptake and phage adsorption and is broadly conserved among Enterobacteriaceae,^37,73,74^ which may explain Cit2’s ability to infect multiple genera. The evolutionary preference of phages for essential or conserved receptors such as FhuA or LPS may provide a selective advantage in accessing hosts.^75^ However, even subtle alterations in receptor-binding sites can disrupt phage adsorption. Previous studies have shown that small deletions in surface-exposed loops of FhuA, particularly within the gating region, significantly alter phage susceptibility without affecting protein localization.^74,76,77^ Although Cit2’s RBP is highly homologous to that of T5, it failed to infect the tested *E. coli* strains, including *E. coli* K12—a strain typically susceptible to T5.^78^ Structural or conformational differences in the FhuA receptor among strains of *E. coli* and *Salmonella* may account for these discrepancies, restricting Cit2’s host range despite RBP homology.

Moreover, although a compatible RBP is a prerequisite for infection, successful adsorption, DNA injection, replication, and lysis also depend on the physiological state and genetic background of the bacterial host.^76,79^ This aligns with findings by Gencay et al., who showed that receptor identity and phage genus together explain up to 79% of host range variance, leaving the remaining variance likely influenced by other factors such as receptor accessibility, intracellular defense mechanisms (e.g., CRISPR-Cas, RM systems), or replication incompatibilities.^59^ The authors also observed that phages that recognize conserved outer membrane proteins, such as T5virus members targeting FhuA or BtuB, tend to have broader host ranges and are commonly isolated from ecologically complex environments like sewage. This correlates with the broad host range and sewage origin of Cit2, supporting the notion that ecological niche and receptor specificity coinfluence host range.

Lateral tail fibers (Ltfs) in T5-like phages play a critical role in initial reversible adsorption by recognizing O-antigens before irreversible engagement with the primary receptor (e.g., FhuA via Pb5), although are not always essential for infection.^58^ Variation in Ltf structure can additionally explain host range differences even among phages that share the same terminal receptor.^59^ Moreover, because O-antigen modifications often incur little or no fitness cost, they offer bacteria an efficient means of evading phage infection.^80^ The synteny analysis of Cit2 revealed substantial variability in Ltf-encoding regions relative to those of other Tequintaviruses, which may help explain differences in host specificity. Additionally, the phylogenetic analysis of the superinfection exclusion Llp protein from Cit2 revealed a divergent clustering pattern, where the Cit2 RBP grouped closely with phage T5, while its Llp clustered with phages that typically infect via BtuB. This divergence may reflect functional adaptations in superinfection exclusion systems and suggests a decoupling between RBP-based host recognition and exclusion mechanisms, potentially influencing the host range spectrum.^81^

The MOI assay demonstrated that Cit2 effectively inhibited the growth of *S.* Enteritidis across a range of phage-to-bacteria ratios, supporting its potential as a biocontrol agent. Although all phage-treated groups exhibited significant bacterial suppression, the timing and extent of growth inhibition varied depending on MOI. At lower MOIs (0.01 and 0.1), limited initial phage numbers allowed early bacterial growth before replication-induced lysis triggered a temporary decline, followed by regrowth and eventual stabilization. Higher MOIs (1–1000) suppressed bacterial growth immediately, but cultures resumed growth after ∼1 h 30 min, stabilizing after 7 h. Despite these differences, final bacterial densities were statistically similar across all MOIs, highlighting Cit2’s consistent efficacy and practical utility in settings where precise dosing may be challenging.^82^ These findings also align with previous reports indicating that low MOIs can still lead to effective bacterial control over time, provided that the phage can replicate efficiently within the host.^83^ Conversely, the transient regrowth observed at higher MOIs may reflect lysis-from-without phenomena or early depletion of susceptible bacterial subpopulations, followed by the expansion of less sensitive clones. Overall, this experiment demonstrates the robustness of Cit2 against *S.* Enteritidis and provides insights into optimizing the phage dosage for potential application in food safety strategies.

Stability assays confirmed that Cit2 remains infective under a broad range of temperatures and pH values, including pH 4–12 and temperatures up to 60 °C. Its resilience to thermal and pH stress highlights its suitability for food industry applications, where fluctuations in temperature and pH may influence phage stability during processing, handling, and storage. Importantly, Cit2 retained significant lytic activity at refrigeration temperatures (4 °C), and reduction in *S.* Enteritidis levels was observed at high MOIs, suggesting the occurrence of “lysis from without”—a valuable mechanism in cold-chain scenarios.^84^ This phenomenon refers to phage-induced bacterial lysis that occurs without replication, triggered instead by the simultaneous binding of numerous phage particles to the bacterial surface.^67^ The resulting structural damage compromises membrane integrity, leading to cell death even under conditions where phage replication is limited, such as at 4 °C. In refrigerated environments where bacterial metabolic activity is reduced, “lysis from without” becomes particularly advantageous, enabling immediate reductions in bacterial counts despite the absence of a productive infection cycle. Beyond its cold-chain relevance, the stability of Cit2 at refrigeration and moderate temperatures reinforces its applicability in real processing environments, including postchilling steps in poultry plants, surface treatments for fresh produce, and incorporation into spray or wash systems without requiring special handling conditions. This robustness increases the likelihood of maintaining effective titers throughout storage, transport, and application, directly supporting its feasibility as a practical biocontrol tool in industrial settings.

Although Cit2 shows strong potential as a biocontrol agent, several challenges inherent to phage-based interventions in food systems must be considered. One important limitation is the possibility of bacterial resistance emergence, a well-documented outcome of phage–bacteria coevolution that may reduce long-term efficacy.^85^ This risk can be mitigated but not entirely eliminated through the use of polyvalent phages or cocktails formulated to target multiple receptors.^86–88^ In addition, phage performance is strongly influenced by food matrix characteristics, including surface topography, moisture, and organic matter, which can hinder phage–host contact and reduce adsorption efficiency.^89–91^ Environmental conditions such as pH, ionic strength, and temperature may also modulate phage activity depending on the application context.^92,93^ Regulatory hurdles remain another key challenge, as approval pathways for phage-based products vary across regions, and requirements for genome safety, manufacturing processes, and environmental impact assessments remain under development.^94,95^ Addressing these limitations will require continued refinement of formulation strategies, including encapsulation, immobilization, and controlled-release approaches, as well as advances in regulatory frameworks to support broader industrial adoption of phage biocontrol tools.

In food models, Cit2 effectively reduced the *S.* Enteritidis counts on both lettuce and chicken meat. Reductions were more pronounced at room temperature on lettuce, consistent with previous studies showing that phage efficacy is matrix- and temperature-dependent.^48,96^ Several previous studies evaluating *Salmonella* control in food matrices used phage cocktails, often at high MOI. For example, Spricigo et al.^48^ used a cocktail of three phages (UAB_Phi20, UAB_Phi78, and UAB_Phi87) at 4 °C and observed reductions of 2.2 and 0.9 log_10_ CFU/g for *S.* Typhimurium and *S.* Enteritidis on chicken meat, respectively, after 7 days and up to 3.9 log_10_ CFU/g on lettuce after 60 min. Pelyuntha and Vongkamjan^49^ also reported reductions in *Salmonella* loads at 4 °C using phage cocktails on chicken meat, employing an MOI of 100 and an evaluation period of 3 days, and observed reductions of 0.3–0.4 log compared to the control between 24 and 72 h of storage. In contrast, the present study used Cit2 as a single phage and achieved comparable reductions after 48 h at 4 °C—conditions under which both bacterial replication and phage activity are naturally limited.

Although the reductions observed were not as high as those reported for phage cocktails, they are still considered significant, particularly given the complexity of chicken meat as a food matrix. The heterogeneous composition of this substrate, including fat, proteins, and irregular surfaces, can hinder phage diffusion and reduce the likelihood of direct interaction with bacterial cells.^97^ This may partially explain the moderate reductions observed. Nonetheless, these findings represent a meaningful impact on the reduction of this pathogen in poultry products and support the potential of Cit2 as a viable biocontrol agent. Additionally, the consistent bacterial suppression observed on chicken meat throughout the 48 h period underscores the phage’s robustness in cold-chain applications. Future work should focus on optimizing the multiplicity of infection (MOI) and strategically combining Cit2 with complementary phages to potentiate antimicrobial efficacy through synergistic interactions. Importantly, future cocktail formulations using Cit2 should consider the phage genus and receptor specificity of the additional phages, as superinfection exclusion systems encoded by Cit2 may inhibit the activity of genetically similar phages that target the same receptor. Therefore, a rational design based on complementary host recognition profiles is recommended. Moreover, the synergistic potential of such cocktails should be carefully evaluated both in vitro and in different food matrices to ensure efficacy across varied industrial scenarios.

In poultry processing plants in Brazil, immersion chilling is the standard method used to reduce microbial loads on carcasses, as outlined by Portaria n° 210/1998 of the Ministério da Agricultura e do Abastecimento.^98^ This process involves the use of cold, potable water (≤4 °C) in countercurrent systems. While effective, it can allow cross-contamination between carcasses. The application of phages as a postchilling spray or dip could serve as a complementary strategy, enhancing *Salmonella* control without altering the sensory properties of the meat or requiring changes to existing workflows. Our results demonstrated that even a short exposure time (10 min) to Cit2 was sufficient to reduce *S.* Enteritidis levels on chicken meat, likely due to the phage’s high adsorption efficiency. This characteristic is particularly advantageous in industrial environments, where carcasses spend limited time in each processing step, making rapid antimicrobial action a key requirement for practical implementation.

Taken together, the findings of this study provide strong evidence supporting the use of Cit2 as a versatile and robust biocontrol agent against *S. enterica*, particularly in the context of poultry products. Its broad host range, lytic efficacy, genomic safety, and environmental stability underscore its suitability for integration into food safety protocols, especially during critical control points such as postchilling stages. While further studies are needed to validate its effectiveness under industrial-scale conditions and in combination with other phages or antimicrobials, the current results establish Cit2 as a promising foundation for the development of targeted phage-based interventions in the control of foodborne pathogens.

## 5 Conclusions

In this section, we isolated and characterized the phage Cit2, a lytic phage belonging to the *Tequintavirus* genus, isolated using *C. freundii* as a host. Host range analysis confirmed the polyvalent nature of Cit2, with lytic activity against *C. freundii*, *S. flexneri*, and different *S. enterica* serovars. This broad host range highlights its potential for applications in food safety, targeting various enteric pathogens.

Morphological analysis revealed typical features of *Tequintavirus*, with an icosahedral head and a long, flexible tail. Genomic analysis confirmed a genome of 112 kb encoding 146 ORFs and 21 tRNAs, with no genes related to lysogeny, virulence, or antimicrobial resistance, reinforcing its genetic safety for biocontrol applications. Phylogenetic and protein analyses suggested that Cit2 likely utilizes FhuA as its primary receptor, similar to phage T5, while its Llp superinfection exclusion protein clustered with phages that use BtuB, possibly reflecting ecological adaptations to reduce competition with other phages.

One-step growth curve assays revealed a short latency period and a high burst size in *C. freundii*, with slightly lower efficiency in *S.* Enteritidis, reflecting differences in host-phage interaction dynamics. Growth kinetics assays with *S.* Enteritidis further demonstrated that higher MOIs led to faster suppression of bacterial growth, particularly in the early hours, while bacterial regrowth was observed after 6–7 h regardless of the MOI. This indicates that both phage dose and bacterial physiological factors influence long-term infection dynamics and bacterial control.

Physicochemical stability tests showed that Cit2 is highly stable across a wide pH range (4–12) and temperatures up to 60 °C, with reduced activity only under extreme conditions such as pH 3 or temperatures above 70 °C. Biocontrol assays in food models demonstrated that Cit2 was able to significantly reduce *S.* Enteritidis counts in both lettuce and chicken meat. Reductions were greater in lettuce at room temperature, while in refrigerated chicken meat the reductions were moderate but sustained over 48 h. These results highlight the influence of the temperature and matrix complexity on phage efficacy.

Additionally, the genomic findings—particularly the absence of lysogeny-, virulence-, and resistance-associated genes, along with receptor-binding features consistent with broad host recognition, strongly support Cit2 as a safe and well-defined candidate for phage-based biocontrol. When considered alongside its broad host range, lytic activity, and environmental stability, these results highlight Cit2’s practical potential for controlling *S. enterica* in food products. Although further validation under industrial-scale conditions and in combination with additional phages is warranted, the evidence presented here establishes Cit2 as a promising tool for targeted phage biocontrol strategies in food safety.

## References

[1] Nadi Z. R., Salehi T. Z., Tamai I. A., Foroushani A. R., Sillanpaa M., Dallal M. M. S. (2020). Evaluation of
Antibiotic Resistance
and Prevalence of Common *Salmonella enterica* Serovars Isolated from Foodborne Outbreaks. Microchem. J..

[2] Brasil . Ministério da Saúde. Secretaria de Vigilância em Saúde Distribuição Temporal Dos Surtos Notificados de Doenças Transmitidas Por Alimentos – Brasil, 2007–2015, 2020; Vol. 51, pp 16–31.

[3] World Health Organization . WHO Estimates of the Global Burden of Foodborne Diseases: Foodborne Disease Burden Epidemiology Reference Group 2007–2015. https://www.who.int/publications/i/item/9789241565165 (accessed May 1, 2025).

[4] Ferrari R. G., Rosario D. K. A., Cunha-Neto A., Mano S. B., Figueiredo E. E. S., Conte-Junior C. A. (2019). Worldwide
Epidemiology of *Salmonella* Serovars in Animal-Based
Foods: A Meta-Analysis. Appl. Environ. Microbiol..

[5] Mechesso A. F., Moon D. C., Kim S.-J., Song H.-J., Kang H. Y., Na S. H., Choi J.-H., Kim H.-Y., Yoon S.-S., Lim S.-K. (2020). Nationwide Surveillance on Serotype Distribution and
Antimicrobial Resistance Profiles of Non-Typhoidal Salmonella Serovars
Isolated from Food-Producing Animals in South Korea. Int. J. Food Microbiol..

[6] Tack D. M., Marder E. P., Griffin P. M., Cieslak P. R., Dunn J., Hurd S., Scallan E., Lathrop S., Muse A., Ryan P. (2019). Preliminary
Incidence and Trends of Infections with
Pathogens Transmitted Commonly Through Food—Foodborne Diseases
Active Surveillance Network, 10 U.S. Sites, 2015–2018. MMWR Morb. Mortal. Wkly. Rep..

[7] Nair, D. V. T. ; Kollanoor Johny, A. Salmonella in Poultry Meat Production. In Food Safety in Poultry Meat Production; Springer International Publishing: Cham, 2019; pp 1–24.

[8] Founou L. L., Founou R. C., Essack S. Y. (2016). Antibiotic Resistance
in the Food
Chain: A Developing Country-Perspective. Front.
Microbiol..

[9] Marchello C. S., Birkhold M., Crump J. A., Martin L. B., Ansah M. O., Breghi G., Canals R., Fiorino F., Gordon M. A., Kim J.-H. (2022). Complications
and Mortality of Non-Typhoidal Salmonella
Invasive Disease: A Global Systematic Review and Meta-Analysis. Lancet Infect. Dis..

[10] Schoch C. L., Ciufo S., Domrachev M., Hotton C. L., Kannan S., Khovanskaya R., Leipe D., Mcveigh R., O’Neill K., Robbertse B. (2020). NCBI Taxonomy: A Comprehensive Update on Curation,
Resources and Tools. Database.

[11] Wessels K., Rip D., Gouws P. (2021). Salmonella in Chicken Meat: Consumption, Outbreaks,
Characteristics, Current Control Methods and the Potential of Bacteriophage
Use. Foods.

[12] Lu J., Wu H., Wu S., Wang S., Fan H., Ruan H., Qiao J., Caiyin Q., Wen M. (2025). Salmonella: Infection
Mechanism and Control Strategies. Microbiol.
Res..

[13] Galán-Relaño Á., Valero
Díaz A., Huerta Lorenzo B., Gómez-Gascón L., Mena Rodríguez M. Á., Carrasco Jiménez E., Pérez Rodríguez F., Astorga Márquez R. J. (2023). Salmonella
and Salmonellosis: An
Update on Public Health Implications and Control Strategies. Animals.

[14] Agência Nacional de Vigilância Sanitária - ANVISA Instrução Normativa - IN No 161, de 1 ° de Julho de 2022. https://www.in.gov.br/en/web/dou/-/instrucao-normativa-in-n-161-de-1-de-julho-de-2022-413366880 (accessed May 1, 2025).

[15] Agência Nacional de Vigilância Sanitária - ANVISA Resolução de Diretoria Colegiada - RDC No 724, de 1 ° de Julho de 2022. https://www.in.gov.br/en/web/dou/-/resolucao-rdc-n-724-de-1-de-julho-de-2022-413364812 (accessed May 1, 2025).

[16] Sorbo A., Pucci E., Nobili C., Taglieri I., Passeri D., Zoani C. (2022). Food Safety Assessment:
Overview of Metrological Issues and Regulatory
Aspects in the European Union. Separations.

[17] Williams M. S., Ebel E. D., Golden N. J., Saini G., Nyirabahizi E., Clinch N. (2022). Assessing the Effectiveness
of Performance Standards
for Salmonella Contamination of Chicken Parts. Int. J. Food Microbiol..

[18] Chiozzi V., Agriopoulou S., Varzakas T. (2022). Advances, Applications, and Comparison
of Thermal (Pasteurization, Sterilization, and Aseptic Packaging)
against Non-Thermal (Ultrasounds, UV Radiation, Ozonation, High Hydrostatic
Pressure) Technologies in Food Processing. Appl.
Sci..

[19] Yang Y., Mikš-Krajnik M., Zheng Q., Lee S.-B., Lee S.-C., Yuk H.-G. (2016). Biofilm
Formation of Salmonella Enteritidis under Food-Related
Environmental Stress Conditions and Its Subsequent Resistance to Chlorine
Treatment. Food Microbiol..

[20] Slattery M., Garvey M. (2025). Chlorine Disinfection
Byproducts: A Public Health Concern
Associated with Dairy Food Contamination. Dairy.

[21] Alonso V. P. P., Furtado M. M., Iwase C. H. T., Brondi-Mendes J. Z., Nascimento M. D. S. (2024). Microbial
Resistance to Sanitizers in the Food Industry:
Review. Crit. Rev. Food Sci. Nutr..

[22] Dubey P., Singh A., Yousuf O. (2022). Ozonation:
An Evolving Disinfectant
Technology for the Food Industry. Food Bioprocess
Technol..

[23] Chacha J. S., Zhang L., Ofoedu C. E., Suleiman R. A., Dotto J. M., Roobab U., Agunbiade A. O., Duguma H. T., Mkojera B. T., Hossaini S. M. (2021). Revisiting Non-Thermal Food Processing and
Preservation Methods—Action Mechanisms, Pros and Cons: A Technological
Update (2016–2021). Foods.

[24] Koutsoumanis K., Alvarez-Ordóñez A., Bolton D., Bover-Cid S., Chemaly M., Davies R., De Cesare A., Herman L., Hilbert F., Lindqvist R. (2022). The Efficacy and Safety of High-pressure Processing of Food. EFSA J..

[25] Tacconelli E., Carrara E., Savoldi A., Harbarth S., Mendelson M., Monnet D. L., Pulcini C., Kahlmeter G., Kluytmans J., Carmeli Y. (2018). Discovery,
Research,
and Development of New Antibiotics: The WHO Priority List of Antibiotic-Resistant
Bacteria and Tuberculosis. Lancet Infect. Dis..

[26] Becker D., Selbach M., Rollenhagen C., Ballmaier M., Meyer T. F., Mann M., Bumann D. (2006). Robust Salmonella
Metabolism
Limits Possibilities for New Antimicrobials. Nature.

[27] Endersen L., Coffey A. (2020). The Use of Bacteriophages for Food Safety. Curr. Opin. Food Sci..

[28] Colom J., Cano-Sarabia M., Otero J., Cortés P., Maspoch D., Llagostera M. (2015). Liposome-Encapsulated Bacteriophages
for Enhanced Oral Phage Therapy against Salmonella Spp. Appl. Environ. Microbiol..

[29] Sillankorva S. M., Oliveira H., Azeredo J. (2012). Bacteriophages
and Their Role in
Food Safety. Int. J. Microbiol..

[30] Mosimann S., Desiree K., Ebner P. (2021). Efficacy of
Phage Therapy in Poultry:
A Systematic Review and Meta-Analysis. Poult.
Sci..

[31] Goodridge L. D., Bisha B. (2011). Phage-Based Biocontrol
Strategies to Reduce Foodborne Pathogens in
Foods. Bacteriophage.

[32] Clavijo V., Morales T., Vives-Flores M. J., Reyes Muñoz A. (2022). The Gut Microbiota
of Chickens in a Commercial Farm Treated with a Salmonella Phage Cocktail. Sci. Rep..

[33] Duc H. M., Son H. M., Yi H. P. S., Sato J., Ngan P. H., Masuda Y., Honjoh K., Miyamoto T. (2020). Isolation,
Characterization
and Application of a Polyvalent Phage Capable of Controlling Salmonella
and *Escherichia coli* O157:H7 in Different
Food Matrices. Food Res. Int..

[34] Li M., Lin H., Khan M. N., Wang J., Kong L. (2014). Effects of Bacteriophage
on the Quality and Shelf Life of *Paralichthys olivaceus* during Chilled Storage. J. Sci. Food Agric..

[35] Guo Y., Li J., Islam M. S., Yan T., Zhou Y., Liang L., Connerton I. F., Deng K., Li J. (2021). Application of a Novel
Phage VB_SalS-LPSTLL for the Biological Control of Salmonella in Foods. Food Res. Int..

[36] Yang S., Sadekuzzaman M., Ha S.-D. (2017). Treatment with Lauric
Arginate Ethyl
Ester and Commercial Bacteriophage, Alone or in Combination, Inhibits
Listeria Monocytogenes in Chicken Breast Tissue. Food Control.

[37] Vasquez I., Retamales J., Parra B., Machimbirike V., Robeson J., Santander J. (2023). Comparative Genomics of a Polyvalent
Escherichia-Salmonella Phage Fp01 and In Silico Analysis of Its Receptor
Binding Protein and Conserved Enterobacteriaceae Phage Receptor. Viruses.

[38] Sukumaran A. T., Nannapaneni R., Kiess A., Sharma C. S. (2015). Reduction of Salmonella
on Chicken Meat and Chicken Skin by Combined or Sequential Application
of Lytic Bacteriophage with Chemical Antimicrobials. Int. J. Food Microbiol..

[39] Reyneke B., Havenga B., Waso-Reyneke M., Khan S., Khan W. (2024). Benefits and
Challenges of Applying Bacteriophage Biocontrol in the Consumer Water
Cycle. Microorganisms.

[40] Ross A., Ward S., Hyman P. (2016). More Is Better:
Selecting for Broad
Host Range Bacteriophages. Front. Microbiol..

[41] Villarroel J., Kleinheinz K., Jurtz V., Zschach H., Lund O., Nielsen M., Larsen M. (2016). HostPhinder: A Phage
Host Prediction
Tool. Viruses.

[42] Van Twest, R. ; Kropinski, A. M. Bacteriophage Enrichment from Water and Soil. In Bacteriophages: Methods and Protocols, Volume 1: Isolation, Characterization, and Interactions; Humana Press: Totowa, NJ, 2009; pp 15–21. 10.1007/978-1-60327-164-6_2 19066806

[43] Adams, M. H. Bacteriophages; Interscience Publishers, 1959.

[44] Sambrook, J. ; Russell, D. W. Molecular Cloning: A Laboratory Manual, 3rd ed.; Cold Spring Harbor Laboratory Press, 2001; Vol. 1.

[45] Khan
Mirzaei M., Nilsson A. S. (2015). Isolation of Phages for Phage Therapy:
A Comparison of Spot Tests and Efficiency of Plating Analyses for
Determination of Host Range and Efficacy. PLoS
One.

[46] Niu Y. D., Stanford K., Kropinski A. M., Ackermann H.-W., Johnson R. P., She Y.-M., Ahmed R., Villegas A., McAllister T. A. (2012). Genomic, Proteomic and Physiological
Characterization
of a T5-like Bacteriophage for Control of Shiga Toxin-Producing *Escherichia coli* O157:H7. PLoS
One.

[47] Sváb D., Falgenhauer L., Rohde M., Szabó J., Chakraborty T., Tóth I. (2018). Identification and Characterization
of T5-Like Bacteriophages Representing Two Novel Subgroups from Food
Products. Front. Microbiol..

[48] Spricigo D. A., Bardina C., Cortés P., Llagostera M. (2013). Use of a Bacteriophage
Cocktail to Control Salmonella in Food and the Food Industry. Int. J. Food Microbiol..

[49] Pelyuntha W., Vongkamjan K. (2022). Combined Effects
of Salmonella Phage Cocktail and Organic
Acid for Controlling Salmonella Enteritidis in Chicken Meat. Food Control.

[50] Camacho C., Coulouris G., Avagyan V., Ma N., Papadopoulos J., Bealer K., Madden T. L. (2009). BLAST+: Architecture and Applications. BMC Bioinf..

[51] Zankari E., Allesøe R., Joensen K. G., Cavaco L. M., Lund O., Aarestrup F. M. (2017). PointFinder: A Novel Web Tool for
WGS-Based Detection
of Antimicrobial Resistance Associated with Chromosomal Point Mutations
in Bacterial Pathogens. J. Antimicrob. Chemother..

[52] Bortolaia V., Kaas R. S., Ruppe E., Roberts M. C., Schwarz S., Cattoir V., Philippon A., Allesoe R. L., Rebelo A. R., Florensa A. F. (2020). ResFinder
4.0 for Predictions of Phenotypes
from Genotypes. J. Antimicrob. Chemother..

[53] Malberg
Tetzschner A. M., Johnson J. R., Johnston B. D., Lund O., Scheutz F. (2020). *In Silico* Genotyping of Escherichia
coli Isolates for Extraintestinal Virulence Genes by Use of Whole-Genome
Sequencing Data. J. Clin. Microbiol..

[54] Joensen K. G., Scheutz F., Lund O., Hasman H., Kaas R. S., Nielsen E. M., Aarestrup F. M. (2014). Real-Time Whole-Genome Sequencing
for Routine Typing, Surveillance, and Outbreak Detection of Verotoxigenic *Escherichia coli*. J. Clin.
Microbiol..

[55] Flayhan A., Wien F., Paternostre M., Boulanger P., Breyton C. (2012). New Insights into Pb5, the Receptor Binding Protein
of Bacteriophage T5, and Its Interaction with Its *Escherichia
coli* Receptor FhuA. Biochimie.

[56] Braun V., Killmann H., Herrmann C. (1994). Inactivation
of FhuA at the Cell
Surface of *Escherichia coli* K-12 by
a Phage T5 Lipoprotein at the Periplasmic Face of the Outer Membrane. J. Bacteriol..

[57] Decker K., Krauel V., Meesmann A., Heller K. J. (1994). Lytic Conversion
of *Escherichia coli* by Bacteriophage
T5: Blocking of the FhuA Receptor Protein by a Lipoprotein Expressed
Early during Infection. Mol. Microbiol..

[58] Heller K., Braun V. (1982). Polymannose O-Antigens
of *Escherichia coli*, the Binding Sites
for the Reversible Adsorption of Bacteriophage
T5+ via the L-Shaped Tail Fibers. J. Virol..

[59] Gencay Y. E., Gambino M., Prüssing T. F., Brøndsted L. (2019). The Genera
of Bacteriophages and Their Receptors Are the Major Determinants of
Host Range. Environ. Microbiol..

[60] Golomidova A. K., Kulikov E. E., Prokhorov N. S., Guerrero-Ferreira R. C., Ksenzenko V. N., Tarasyan K. K., Letarov A. V. (2015). Complete
Genome
Sequences of T5-Related *Escherichia coli* Bacteriophages DT57C and DT571/2 Isolated from Horse Feces. Arch. Virol..

[61] Mondigler M., Ayoub A. T., Heller K. J. (2006). The DNA Region of
Phage BF23 Encoding
Receptor Binding Protein and Receptor Blocking Lipoprotein Lacks Homology
to the Corresponding Region of Closely Related Phage T5. J. Basic Microbiol..

[62] Bradbeer C., Woodrow M. L., Khalifah L. I. (1976). Transport of Vitamin B12 in *Escherichia coli*: Common Receptor System for Vitamin
B12 and Bacteriophage BF23 on the Outer Membrane of the Cell Envelope. J. Bacteriol..

[63] Rabsch W., Ma L., Wiley G., Najar F. Z., Kaserer W., Schuerch D. W., Klebba J. E., Roe B. A., Gomez J. A. L., Schallmey M. (2007). FepA- and TonB-Dependent Bacteriophage H8: Receptor Binding and Genomic
Sequence. J. Bacteriol..

[64] Braun V., Schaller K., Wolff H. (1973). A Common Receptor
Protein for Phage
T5 and Colicin M in the Outer Membrane of *Escherichia
coli* B. Biochim. Biophys. Acta,
Biomembr..

[65] Kim M., Ryu S. (2011). Characterization
of a T5-Like Coliphage, SPC35, and Differential
Development of Resistance to SPC35 in *Salmonella enterica* Serovar Typhimurium and *Escherichia coli*. Appl. Environ. Microbiol..

[66] Golomidova A., Kulikov E., Prokhorov N., Guerrero-Ferreira R., Knirel Y., Kostryukova E., Tarasyan K., Letarov A. (2016). Branched Lateral
Tail Fiber Organization in T5-Like Bacteriophages DT57C and DT571/2
Is Revealed by Genetic and Functional Analysis. Viruses.

[67] Abedon S.
T. (2011). Lysis from
Without. Bacteriophage.

[68] Sukjoi C., Buddhasiri S., Tantibhadrasapa A., Kaewsakhorn T., Phothaworn P., Nale J. Y., Lopez-Garcia A. V., AbuOun M., Anjum M. F., Malik D. J. (2022). Therapeutic
Effects of Oral Administration of Lytic Salmonella Phages in a Mouse
Model of Non-Typhoidal Salmonellosis. Front.
Microbiol..

[69] Manohar P., Loh B., Athira S., Nachimuthu R., Hua X., Welburn S. C., Leptihn S. (2020). Secondary
Bacterial Infections During Pulmonary Viral
Disease: Phage Therapeutics as Alternatives to Antibiotics?. Front. Microbiol..

[70] Costa M. J., Pastrana L. M., Teixeira J. A., Sillankorva S. M., Cerqueira M. A. (2023). Bacteriophage Delivery Systems for
Food Applications:
Opportunities and Perspectives. Viruses.

[71] Bailly-Bechet M., Vergassola M., Rocha E. (2007). Causes for the Intriguing Presence
of TRNAs in Phages. Genome Res..

[72] Bonhivers M., Ghazi A., Boulanger P., Letellier L. (1996). FhuA, a Transporter
of the *Escherichia coli* Outer Membrane,
Is Converted into a Channel upon Binding of Bacteriophage T5. EMBO J..

[73] Wang Y., Chen X., Hu Y., Zhu G., White A. P., Köster W. (2018). Evolution and Sequence Diversity
of FhuA in Salmonella
and Escherichia. Infect. Immun..

[74] Killmann H., Herrmann C., Wolff H., Braun V. (1998). Identification of a
New Site for Ferrichrome Transport by Comparison of the FhuA Proteins
of *Escherichia coli*, *Salmonella paratyphi* B, *Salmonella
typhimurium*, and *Pantoea agglomerans*. J. Bacteriol..

[75] Chaturongakul S., Ounjai P. (2014). Phage–Host Interplay:
Examples from Tailed Phages
and Gram-Negative Bacterial Pathogens. Front.
Microbiol..

[76] Labrie S. J., Samson J. E., Moineau S. (2010). Bacteriophage
Resistance Mechanisms. Nat. Rev. Microbiol..

[77] Braun M., Killmann H., Braun V. (1999). The B-barrel
Domain of FhuAΔ5–160
Is Sufficient for TonB-dependent FhuA Activities of *Escherichia coli*. Mol. Microbiol..

[78] Carmel G., Coulton J. W. (1991). Internal Deletions
in the FhuA Receptor of *Escherichia coli* K-12 Define Domains of Ligand Interactions. J. Bacteriol..

[79] Egido J. E., Costa A. R., Aparicio-Maldonado C., Haas P.-J., Brouns S. J. J. (2022). Mechanisms
and Clinical Importance of Bacteriophage Resistance. FEMS Microbiol. Rev..

[80] Kim M., Ryu S. (2012). Spontaneous and transient
defence against bacteriophage by phase-variable
glucosylation of O-antigen in *Salmonella enterica* serovar Typhimurium. Mol. Microbiol..

[81] Haggård-Ljungquist E., Halling C., Calendar R. (1992). DNA Sequences of the Tail Fiber Genes
of Bacteriophage P2: Evidence for Horizontal Transfer of Tail Fiber
Genes among Unrelated Bacteriophages. J. Bacteriol..

[82] Abedon S. T. (2016). Phage Therapy
Dosing: The Problem(s) with Multiplicity of Infection (MOI). Bacteriophage.

[83] Hyman, P. ; Abedon, S. T. Bacteriophage Host Range and Bacterial Resistance. In Advances in Applied Microbiology, 2010; pp 217–248. 10.1016/S0065-2164(10)70007-1 20359459

[84] Azari R., Yousefi M. H., Taghipour Z., Wagemans J., Lavigne R., Hosseinzadeh S., Mazloomi S. M., Vallino M., Khalatbari-Limaki S., Berizi E. (2023). Application of the Lytic Bacteriophage Rostam to Control
Salmonella Enteritidis in Eggs. Int. J. Food
Microbiol..

[85] Oechslin F. (2018). Resistance
Development to Bacteriophages Occurring during Bacteriophage Therapy. Viruses.

[86] Yoo S., Lee K.-M., Kim N., Vu T. N., Abadie R., Yong D. (2024). Designing Phage Cocktails to Combat the Emergence of Bacteriophage-Resistant
Mutants in Multidrug-Resistant *Klebsiella pneumoniae*. Microbiol. Spectrum.

[87] Vaitekenas A., Tai A. S., Ramsay J. P., Stick S. M., Kicic A. (2021). Pseudomonas
Aeruginosa Resistance to Bacteriophages and Its Prevention by Strategic
Therapeutic Cocktail Formulation. Antibiotics.

[88] Oromí-Bosch A., Antani J. D., Turner P. E. (2023). Developing
Phage Therapy That Overcomes
the Evolution of Bacterial Resistance. Annu.
Rev. Virol..

[89] Ranveer S. A., Dasriya V., Ahmad M. F., Dhillon H. S., Samtiya M., Shama E., Anand T., Dhewa T., Chaudhary V., Chaudhary P. (2024). Positive and Negative Aspects of Bacteriophages
and Their Immense Role in the Food Chain. npj
Sci. Food.

[90] Efenberger-Szmechtyk M., Nowak A. (2025). Bacteriophage Power: Next-Gen Biocontrol Strategies for Safer Meat. Molecules.

[91] Shannon R., Radford D. R., Balamurugan S. (2020). Impacts of
Food Matrix on Bacteriophage
and Endolysin Antimicrobial Efficacy and Performance. Crit. Rev. Food Sci. Nutr..

[92] Huang C., Shi J., Ma W., Li Z., Wang J., Li J., Wang X. (2018). Isolation, Characterization,
and Application of a Novel Specific
Salmonella Bacteriophage in Different Food Matrices. Food Res. Int..

[93] Jończyk E., Kłak M., Międzybrodzki R., Górski A. (2011). The Influence
of External Factors on Bacteriophages—Review. Folia Microbiol..

[94] Yang Q., Le S., Zhu T., Wu N. (2023). Regulations
of Phage Therapy across
the World. Front. Microbiol..

[95] Sulakvelidze, A. ; Pasternack, G. R. Industrial and Regulatory Issues in Bacteriophage Applications in Food Production and Processing. In Bacteriophages in the Control of Food- and Waterborne Pathogens; ASM Press: Washington, DC, USA, 2014; pp 297–326.

[96] Fister S., Robben C., Witte A. K., Schoder D., Wagner M., Rossmanith P. (2016). Influence
of Environmental Factors on Phage–Bacteria
Interaction and on the Efficacy and Infectivity of Phage P100. Front. Microbiol..

[97] O’Flynn G., Ross R. P., Fitzgerald G. F., Coffey A. (2004). Evaluation of a Cocktail
of Three Bacteriophages for Biocontrol of *Escherichia
coli* O157:H7. Appl. Environ.
Microbiol..

[98] Ministério da Agricultura e do Abastecimento Portaria No 210, de 10 de Novembro de 1998: Aprova o Regulamento Técnico Da Inspeção Tecnológica e Higiênico-Sanitária de Carne de Aves. https://www.gov.br/agricultura/pt-br/assuntos/inspecao/produtos-animal/arquivos-portal-carne-aves/PORTARIA210.pdf (accessed April 21, 2025).

